# Growth rate determines prokaryote-provirus network modulated by temperature and host genetic traits

**DOI:** 10.1186/s40168-022-01288-x

**Published:** 2022-06-14

**Authors:** Zhenghua Liu, Qingyun Yan, Chengying Jiang, Juan Li, Huahua Jian, Lu Fan, Rui Zhang, Xiang Xiao, Delong Meng, Xueduan Liu, Jianjun Wang, Huaqun Yin

**Affiliations:** 1 Key Laboratory of Biometallurgy of Ministry of Education, School of Minerals Processing and Bioengineering, Central South University, Changsha, 410006 China; 2 State Key Laboratory of Lake Science and Environment, Nanjing Institute of Geography and Limnology, Chinese Academy of Sciences, Nanjing, 210008 China; 3 Environmental Microbiomics Research Center, School of Environmental Science and Engineering, Sun Yat-sen University, Guangzhou, 510006 China; 4 State Key Laboratory of Microbial Resources, Institute of Microbiology, Chinese Academy of Sciences, Beijing, 100101 China; 5 College of Agronomy, Hunan Agricultural University, Changsha, 410125 China; 6 State Key Laboratory of Microbial Metabolism, School of Life Sciences and Biotechnology, Shanghai Jiao Tong University, Shanghai, 200240 China; 7 Department of Ocean Science and Engineering, Southern University of Science and Technology, Shenzhen, 518055 China; 8 State Key Laboratory of Marine Environmental Science, The Institute of Marine Microbes and Ecospheres, Xiamen University, Xiamen, 361102 China

**Keywords:** Host-virus interaction, Growth rate, Specialization, Temperature, Infection cycle, Genetic traits

## Abstract

**Background:**

Prokaryote-virus interactions play key roles in driving biogeochemical cycles. However, little is known about the drivers shaping their interaction network structures, especially from the host features. Here, we compiled 7656 species-level genomes in 39 prokaryotic phyla across environments globally and explored how their interaction specialization is constrained by host life history traits, such as growth rate.

**Results:**

We first reported that host growth rate indicated by the reverse of minimal doubling time was negatively related to interaction specialization for host in host-provirus network across various ecosystems and taxonomy groups. Such a negative linear growth rate-specialization relationship (GrSR) was dependent on host optimal growth temperature (OGT), and stronger toward the two gradient ends of OGT. For instance, prokaryotic species with an OGT ≥ 40 °C showed a stronger GrSR (Pearson’s *r* = −0.525, *P* < 0.001). Significant GrSRs were observed with the presences of host genes in promoting the infection cycle at stages of adsorption, establishment, and viral release, but nonsignificant with the presence of immune systems, such as restriction-modification systems and CRISPR-Cas systems. Moreover, GrSR strength was increased with the presence of temperature-dependent lytic switches, which was also confirmed by mathematical modeling.

**Conclusions:**

Together, our results advance our understanding of the interactions between prokaryotes and proviruses and highlight the importance of host growth rate in interaction specialization during lysogenization.

Video Abstract

**Supplementary Information:**

The online version contains supplementary material available at 10.1186/s40168-022-01288-x.

## Introduction

The structure of prokaryote-virus interaction networks is closely related to ecosystem functions [1], such as biogeochemical cycles [2]. Specific host-virus pairs in networks are primarily established on the basis of their gene-for-gene coevolution [1, 3], during which host serving as prey are often initiators to constantly update their antivirus (predator) mechanisms for survival [4]. This forwardly evolutionary strategy of hosts not only narrows their spectrum of viral susceptibilities but also enforces viruses to participate in an arms race and become specialists, especially at large phylogenetic scales of host-virus interactions [5]. Such coevolutionary dynamics enhance the heterogeneity of species specialization in cross-infection patterns [1], which contributes to nonrandom networks with nested, modular, or nested-modular structures [5–7]. However, previous studies have focused mostly on the host range or specificity of viruses in exploring specialized host-virus interactions [6–8], while the host side is understudied. Complementary to the findings about viruses, the study of host features such as interaction specialization for host could provide a novel insight into host-virus network structure.

**Fig. 1:**
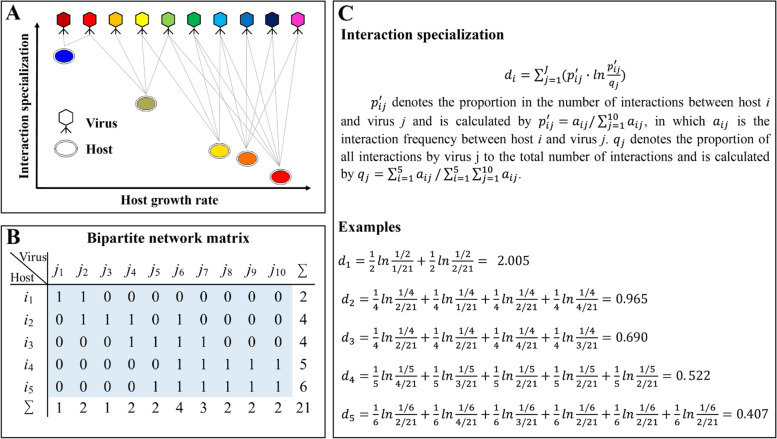
Interaction specialization of host in host-virus network. **A** We hypothesized that interaction specialization for host in host-virus network is negatively related to host growth rate. It is noted that interaction specialization of host comprehensively considers both the number of different viral interactions and host range of viruses. Colors of triangles and circles represent different virus species and host species with gradient growth rates, respectively. **B** Adjacency matrix of host-virus interaction network (blue background) in plot **A**. **C** Formulation of interaction specialization of host in host-virus network, which is calculated by Shannon index *d* (see details in the ‘Methods’ section)

Host specialized on the set of viruses is primarily dependent on the alignments between its physiological status and the viral interests [9]. Such specific matches could lead to the phenomenon of the presence of proviruses in prokaryotic genomes being associated with host life history traits [9, 10], aside from molecular interactions from receptor to immune recognition [11]. The host growth rate could be one of the most important traits in determining host-virus interaction outcomes [12–16], and its high variability across species could result in the heterogeneous distributions of proviruses within certain prokaryotic clades [9]. Following this clue, we proposed that host specialization in host-virus interactions will be linked to the host growth rate based on the three lines of indirect evidences. First, an improved growth rate of the host could be accompanied with a reduced range of resistance to viruses [17, 18], which is partly contributed by the tradeoff between immunity and growth rate [19]. Second, high-host density stimulated by a high-growth rate cloud provide more chances of host cells encountering more virus species and enhances the virus adsorption rate [20], which may increase the probability in coinfection or even superinfection with diverse viruses in the host and generally modelled by Kill-the-Winner dynamic [21]. Third, slow-growing species favoring lysogeny [9] could allow proviruses to evolve competition strategies (e.g., surface modification) in host for inhibiting further viral infection [22]. Inspired by above findings, we hypothesized that prokaryotic species attaining a fast growth rate (fast grower) may show less specificity on virus species than slow growers, that is, growth rate-specialization relationships (GrSRs) (Fig. 1).

We further expect that GrSRs could be influenced by the host growth environment and genetic traits [4, 23, 24]. For example, in high-temperature environment, thermal stress has stronger effects on the host than its viruses [23], which would lead to the host changes in outer-membrane protein expression [25] and immune activities [26]. These host responses alter the host susceptibility or specificity to viral infection, revealing that a temperature-dependent infection structure is primarily established by the host [23]. In saline environments, ironic strength not only affect the host physiological status [23] but also largely determines the infection activity of specialized viruses [27–30], implying the interactions of host-virus pairs could be constrained by environment salinity. Furthermore, host genetic traits are fundamental to their interaction specialization in infection networks during host-virus co-evolution. For instance, host resistance to viruses could shift with the evolution of some genetic traits, including the mutations in surface receptors, such as outer-membrane proteins and flagellum or pilin proteins [31], and the development of diverse immune systems, such as restriction-modification (RM) systems [32] and clustered regularly interspaced short palindromic repeats (CRISPR) and their associated protein (Cas) systems [33]. CRISPR-Cas systems, adaptive immune systems targeting specific viral sequences [34], could play a key role in shaping interaction specialization of host by mediating inefficient viral infection, or selectively inactivating viruses and allowing hosts to employ specific viruses during evolution for adaptation to diverse environments [35]. Despite the guidance of above findings, which and how environmental factors and host life history or genetic traits affect interaction specialization for host in prokaryote–virus networks are largely unknown and worth further investigations at global scale.

Compared with virulent viruses, proviruses as temperate viruses could increase host fitness such as growth rate [36] and temperature tolerance [37] by regulating gene expressions of host cell population at lysogenic state. This suggests that the interactions between host and proviruses are more dependent on host traits and environment factors than virulent viruses, and we therefore focused on the host-provirus interactions to investigate the drivers shaping interaction specialization for host. Here, we compiled 7656 species-level genomes from approximately 170,000 strain-level genomes of bacteria and archaea [38] across 15 ecosystems and 15 phyla and construct a host-provirus bipartite network based on the occurrences of proviruses in host genomes. We used Shannon index *d’* to quantify the interaction specialization for host within host-provirus network (Fig. 1). To uncover the role of host life history and genetic traits playing in its specialization in host-provirus interactions, we considered three main aspects of prokaryotic host: the growth rate estimated by the reverse of the minimal doubling time (*DT*), the optimal growth temperature (OGT), and the genes responsible for the infection cycle. We further established a mathematical model to explore the key determinants contributing to interaction specialization for host from the perspective of population dynamics*.* We had three main aims: (1) How does the host growth rate influences its interaction specialization across ecosystems and taxonomic groups? (2) What environmental factors or (3) genetic traits potentially modulate the effects of host growth rate on interaction specialization? Our results suggest that host growth rate plays a key role in shaping their interaction specialization during lysogenization, which is strengthened by high or low temperature and potentially constrained by molecular processes in the infection cycle.

## Methods

### Features of prokaryotes

We selected 7656 genomes as representative species from approximately 170,000 prokaryote strains complied by Madin et al. [38] and downloaded them from the NCBI according to accession numbers. The filtered genomes included 390 archaea and 7266 bacteria, and their completeness was greater than 90%. We considered species with the maximal genome size at the strain level as representative species. The natural environments were classified according to the protocols of Earth Microbiome Project Ontology (EMPO) [39]. Coding sequences in prokaryote genomes were also detected by Prokka v1.14.6 [40] with default parameters. The minimal doubling times were predicted based on ribosomal protein genes via the “predictGrowth” function in the “gRodon” v1.0.0 package [41], the reverse of which was used to indicate the host growth rate in units of doublings per day. The OGTs were predicted by software Tome v1.0 software [42] using species coding sequences with default parameters. Phylogenetic tree of all prokaryotic species was constructed by GToTree v1.6.12 software [43] based on 25 shared genes for bacteria and archaea (-H Bacteria_and_Archaea) with default parameters.

### Proviruses

Proviruses in prokaryote genomes were identified by Phispy v4.1.22 based on AT and GC skews [44] with default parameters, and prophage genomes with lengths less than 3 kb were removed. To generate viral clusters at genus level, the remaining provirus genomes were clustered and taxonomically assigned by vConTACT2 v0.9.19 [45] against “ProkaryoticViralRefSeq201-Merged” reference via Diamond v0.9.24.125 [46] and ClusterONE (vcontact2 --rel-mode Diamond --pcs-mode MCL --vcs-mode ClusterONE). Provirus coding sequences were clustered by MMseqs2 v12.113e3 [47] with 70% identity and 70% coverage (mmseqs easy-linclust --min-seq-id 0.7 -c 0.7 --cov-mode 1). The completeness of provirus sequences were accessed by Checkv v0.8.1 software [48] with default parameters.

### Surface receptor proteins

Host genes encoding phage protein receptors were searched for by DIAMOND against the phageReceptor database [49] with 50% identity.

### Immunity systems

CRISPR arrays and Cas genes were identified by CRISPR-finder [50]. CRISPR-Cas systems were composed of CRISPR arrays and the closest Cas genes with distances less than 600 bp (bacteria: -cs -ccvr -vi 600; archaea: -ac -ccvr -vi 600). RM systems were identified by DIAMOND against the REBASE database [51] with 50% identity. The pair genes encoding restriction endonucleases and methylase were considered as a restriction-modification system when there were less than 5 genes between this pair genes.

### Lytic switches

Genes encoding the repressors CI and Cro were searched for by DIAMOND against sequences collected from the UniProt database [52] with 50% identity. Genes encoding the repressors FpsA and FpsR were searched for against sequences provided by a previous study [53].

### Species specialization of viral infections

A host-provirus network was constructed according to the coexistence of host and viral clusters. Since the inclusions of singletons and doubletons within host-provirus network would lead to a lot of perfect specialists during calculating interaction specialization *d’*, we removed singletons and doubletons of hosts and viral clusters for downstream analyses. Interaction specialization of host was estimated by the standardized specialization index *d’* at host level within host-provirus network with *I* hosts × *J* viruses, which was carried out by the “dfun” function in the “bipartite” v2.16 package [54]. The value *d’* is derived from Shannon’s diversity index [55], which represents how specialized a given species is in relation to available interacting partners [56]. It is given by following formula:

$${\displaystyle \begin{array}{c}{d}_i={\sum}_{j=1}^J\left({p}_{ij}^{\prime}\bullet \mathit{\ln}\frac{p_{ij}^{\prime }}{q_j}\right)\ \mathrm{and}\\ {}{d}_i^{\prime }=\frac{d_i-{d}_{min}}{d_{max}-{d}_{min}},\end{array}}$$

where ${d}_i^{\prime }$ represents standardized values derived from nonstandardized *d*_*i*_ of host *i*. ${p}_{ij}^{\prime }$ denotes the proportion in the number of interactions between host *i* and virus *j,* calculated by ${p}_{ij}^{\prime }={a}_{ij}/{\sum}_{j=1}^J{a}_{ij}$, in which *a*_*ij*_ is the interaction frequency between host *i* and virus *j* (presence: 1; absence: 0). *q*_*j*_ denotes the proportion of all interactions by virus *j* to the total number of interactions, given by ${q}_j={\sum}_{i=1}^I{a}_{ij}/\sum_{i=1}^I{\sum}_{j=1}^J{a}_{ij}$. The value of ${d}_i^{\prime }$ ranges from 0 for extreme generalization to 1 for extreme specialization.

### Mathematical model of viral infection dynamics regulated by temperature

The presented mathematical model (Fig. S1) is an extended combination of previous works [57, 58]. We considered a spatially homogeneous habitat of unitary volume with a maximum capacity *C*, in which *B*^+^ and *B*^−^ are presented as the population density of nonsusceptible and susceptible cells to viruses, respectively, and *P* is the population density of viruses. For simplicity, we assumed that the growth of cells is described by the logistic function *φ*(*N*) multiplied by temperature fitness *θ*(*T*), where *N* and *T* are the population density of cells and environment temperature, respectively. We assumed that all viruses are ecological similar and adsorb to cells with an adsorption constant rate *δ*. The *B*^−^ infected by *i* viral species is presented as ${B}_i^{-}$, the probability of which is *Pr*(*X* = *i*) and related to population density of cells and viruses. Here, we assumed ${B}_0^{-}$ is lucky fellows escaping viral infection. The fraction *α* of ${B}_i^{-}$ enters into lysogeny, whereas the remaining 1 − *α* release *β*virus particles. The lysogen ${B}_i^{-}$ could be induced to lyse and release *β* viral particles with an induction rate *ξ*(*T*). For lysogen ${B}_i^{-}$, secondary infections are invalid and result in the loss of infecting viruses. The total cell population density is denoted by $N={B}^{+}+{B}^{-}+\sum_{i\ne 0}{B}_i^{-}$. It is noted that host death due to lysis was considered in the term of *B*^−^. We assumed that there is a time delay (latent period) of *τ* between infection and lysis of lysogens.

Given the above assumptions, the model of viral infection dynamics regulated by temperature is given by delay differential equations, as follows:

$${\displaystyle \begin{array}{l}\frac{d{B}^{+}(t)}{dt}=\underset{\mathrm{Cell}\ \mathrm{growth}}{\underbrace{\varphi_{+}\left(N(t)\right){\theta}_{+}(T){B}^{+}(t)}},\\ {}\frac{d{B}^{-}(t)}{dt}=\underset{\mathrm{Cell}\ \mathrm{growth}}{\underbrace{\varphi_{-}\left(N(t)\right){\theta}_{-}(T){B}^{-}(t)}}-\underset{\mathrm{Infecting}\ \mathrm{cell}}{\underbrace{\ \delta {B}^{-}(t)P(t)}}+\underset{\mathrm{Cell}\mathrm{s}\ \mathrm{escaping}\ \mathrm{infection}}{\underbrace{\delta Pr\left(0|P(t),{B}^{-}(t),N(t)\right){B}^{-}(t)P(t)}},\\ {}\begin{array}{l}\frac{d{B}_i^{-}(t)}{dt}=\underset{\mathrm{Cell}\ \mathrm{growth}}{\underbrace{\varphi_{-}\left(N(t)\right){\theta}_{-}(T){B}_i^{-}(t)}}+\underset{\mathrm{Lysogenization}}{\underbrace{\alpha \delta \mathit{\Pr}\left(i|P(t),{B}^{-}(t),N(t)\right){B}^{-}(t)P(t)}}-\underset{\mathrm{Induction}}{\underbrace{\xi (T){B}_i^{-}(t)}},i\ne 0,\\ {}\frac{dP(t)}{dt}=\underset{\mathrm{Lysis}}{\underbrace{\sum_{i\ne 0}\left(1-\alpha \right)\delta \beta Pr\left(i|P(t),{B}^{-}(t),N(t)\right)\ {B}^{-}(t)P\left(\ t\right)}}+\underset{\mathrm{Induction}}{\underbrace{\sum_{i\ne 0}\beta \xi (T){B}_i^{-}(t)}}-\underset{\mathrm{Adsorption}}{\underbrace{\delta N(t)P(t)}},\\ {}\frac{dN(t)}{dt}=\frac{d{B}^{+}(t)}{dt}+\frac{d{B}^{-}(t)}{dt}+\frac{d{B}_i^{-}(t)}{dt}.\end{array}\end{array}}$$

The growth rates of *B*^+^ and *B*^−^ are described by logistic functions of *φ*_+_(*N*(*t*)) = *V*_+_(1 − *N*(*t*)/*C*) and *φ*_−_(*N*(*t*)) = *V*_−_(1 − *N*(*t*)/*C*), respectively. The maximal growth rates of *B*^+^ and *B*^−^ are presented by *V*_+_ and *V*_−_, respectively.

The temperature fitness of *B*^+^ and *B*^−^ is described by Gaussian functions [59] of ${\theta}_{+}(T)=\exp \left(-\frac{{\left(T-{T}_{+}\right)}^2}{2{\sigma}_{+}^2}\right)$ and ${\theta}_{-}(T)=\exp \left(-\frac{{\left(T-{T}_{-}\right)}^2}{2{\sigma}_{-}^2}\right)$, respectively. The OGTs of *B*^+^ and *B*^−^ are presented by *T*_+_ and *T*_−_, respectively. The temperature niche breadths of *B*^+^ and *B*^−^ are presented by *σ*_+_ and *σ*_−_, respectively.

We assumed that host cells are infected by *i* viruses at time *t* occurs as a series of Bernoulli trials with the probability of success equal to *p*. Considering that the number of Bernoulli trials *n* is equal to viral particles and approximately infinite, the probability of a cell infected by *i* viruses at time *t* could be calculated by the Poisson formula and given by $\mathit{\Pr}\left(X=i\right)=\frac{\lambda^i}{i!}{e}^{-\lambda },\lambda = np$. Here, *λ* is the average number of viruses infecting a host cell and fluctuating with the ratio of virus population density (*P*) to total cell population density (*N*). Since merely susceptible cells could be infected by viruses, the average number of viruses infecting a susceptible cell should be multiplied by the ratio of susceptible cell population density *B*^−^ to the total cell population density *N* and formulized by $\lambda =\left({\lambda}_0+\frac{P}{N}\right){B}^{-}/N$. Here, *λ*_0_ is the basic average number of viral species infecting host cell.

The temperature-dependent switch of lysogeny induction is described by a sigmoid function. We considered that there are heat- [60] and cold-activated switches [61], which are formulated by ${f}_h(T)=\frac{1}{1+\exp \left(k\left({T}_h-T\right)\right)}$ and ${f}_c(T)=\frac{1}{1+\exp \left(k\left(T-{T}_c\right)\right)}$. *T*_*h*_ and *T*_*c*_ represent the temperatures of a half of the lysogeny events switching to lysis activated by heat and cold temperature, respectively. The parameter of *k* is a constant. Subsequently, the induction rate is described by *ξ*(*T*) = *ξ*_0_ max(*f*_*h*_(*T*), *f*_*c*_(*T*)), where *ξ*_0_ is the induction rate constant.

Species specialization of host *d* is calculated by the following equation:

$${\displaystyle \begin{array}{c}S=\sum_{i\ne 0}{B}_i^{-}\\ {}d=\frac{1}{\sum_{i\ne 0}\frac{B_i^{-}\times i}{S}}\end{array}}$$

where *S* is the population density of lysogens.

All constant parameters and initial states were listed in Tables S1 and S2, respectively. The system of delay differential equations was solved by Simon Wood’s solv95 program, which was conducted with “PBSddesolve” package [62] in R v3.6 environment [63]. We deposited the codes of the model on Github repository https://github.com/zhenghualiu/GrSRs.

### Statistical analyses

The linear relationships between host growth rate and interaction specialization *d’* (GrSR) were estimated by a linear regression model [64]. To control the effects of phylogenetic structure in linear model for each phylum, phylogenetically corrected linear regression was further conducted by phylolm function with the Brownian motion model (BM) and the Pagel’s *delta* model from “phylolm” v2.6.4 package [65]. For multiple testing analyses, *P* values were adjusted by Benjamini-Hochberg correction [66]. To detect the changes in GrSRs along with the OGT gradient, we used moving window approaches [67, 68] to locate sudden changes in the strength of relationships between host growth rate (log10-scaled) and *d’*. Specifically, dataset was divided into overlapping bins by 10°C (e.g., 10–20°C, 11–21°C, and 12–22°C until 70–80°C) and 20°C moving windows (e.g., 10–30°C, 11–31°C, and 12–32°C until 60–80°C) of OGT between 10 and 80°C. The Poisson distribution of the multiplicity of infections in lysogens was estimated by the maximum-likelihood method [69], and its significance was assessed by goodness-of-fit tests [70]. The above analyses were conducted in the R v3.6 environment [63].

## Results and discussion

In total, we identified 11,798 provirus sequences in 5283 and 166 species-level genomes for bacteria and archaea, respectively. The number of proviruses in genomes followed the Poisson distribution with an average of 1.55 (Fig. S2). The genomic length of provirus ranged from 3.01 to 224.01 kb and averaged 32.94 (± 19.66, SD) kb. We accessed the completeness of provirus genomes by CheckV [48] and found that 2549 sequences had completeness > 50% and 3447 sequences had completeness ≤ 50% while the others could not be determined. Based on gene-sharing network constructed by vContact2 [45], all provirus sequences could be classified into 1581 viral clusters, and 5.88% of which representing 7.65% of provirus sequences could be assigned with order-level taxonomies.

A host–provirus network was constructed based on the occurrence of proviruses in host genomes and used to further quantify interaction specialization of host (see details in the ‘Methods’ section). Interaction specialization of host was determined by the Shannon diversity index *d* and standardized by min-max scaling (*d’*) [71]. It is noted that specialization *d’* (interaction specialization of host) comprehensively considers both the number of different viral interactions and host range of virus, and a higher value of *d’* indicates that host is more specialized to interact with viruses (Fig. 1). Since the singletons and doubletons would introduce 61% of potentially spurious proviruses into host-provirus network due to their lack of genome completeness (Fig. S3). If we included them in the calculation of interaction specialization *d’*, it led to 31% of the most specialized host with an extreme *d’* value of 1 and caused potential bias due to the non-normal distribution of *d’* (Fig. S4A). In contrast, the exclusion of singletons and doubletons could result in an approximately normal distribution of *d’* (Fig. S4B). Thus, to ensure the reliability in downstream analyses, we did not include the singletons and doubletons of viral clusters and host in host-provirus network. Consequently, we got a host–provirus bipartite network of 3115 hosts × 548 viral clusters with 4339 links, where each host species averagely interacted with 1.39 (± 0.68) viral clusters. Across the 39 phyla of bacteria and archaea, specialization *d’* averaged 0.64 (± 0.15) and was significantly correlated with the reverse of viral cluster number, with a weak correlation of −0.045 (df = 3054, *P* = 0.013; Fig. S5). Furthermore, specialization *d’* was significantly correlated with the copy number of the 5S rRNA, 16S rRNA, and 23S rRNA genes (all *P* < 0.001; Fig. S5), which indicates that interaction specialization of host is closely linked to the host growth rate (*r* = −0.119, *P* < 0.001; Fig. S5).

### Host growth rate shapes interaction specialization

**Fig. 2:**
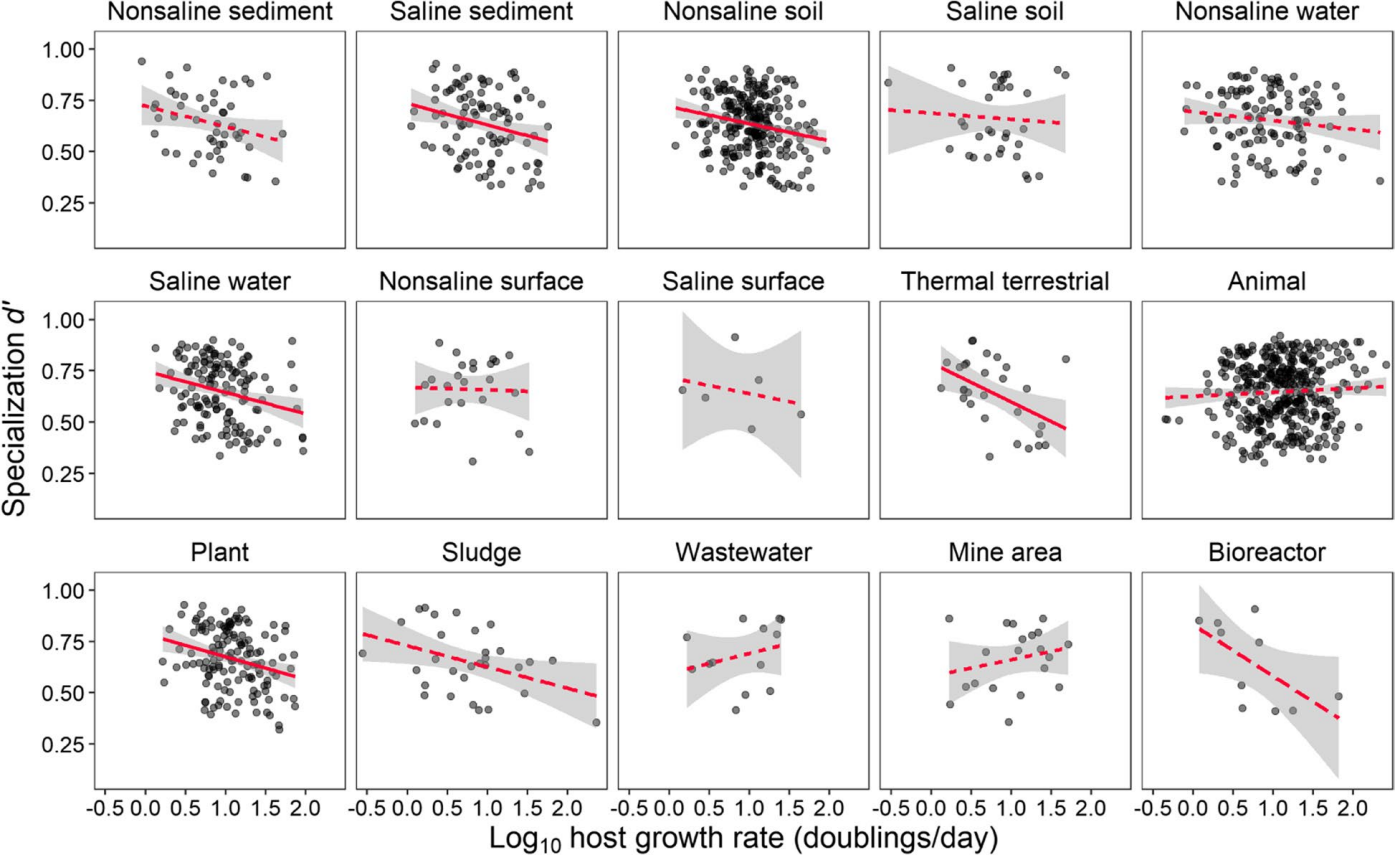
Relationships between host growth rate and their interaction specialization (*d’*) across ecosystems. The host growth rates are log10-scaled. Solid lines denote significant linear GrSRs (all *P*_adj_ < 0.05), while dashed lines are not linear GrSRs

We found that there were significant linear relationships between host growth rate and specialization *d’* across various ecosystems (Figs. 2 and S6) and phyla (Fig. S7). For instance, the negative linear growth rate-specialization relationships (GrSRs) were observed in natural environments such as terrestrial thermal systems (*R*^2^_adj_ = 0.19, *P* < 0.05; Figs. 2 and S6). There was also significantly negative GrSRs in various phyla, such as Deinococcus-Thermus (*R*^2^_adj_ = 0.468, *P* < 0.05; Fig. S7, Table S3). Notably, one exception of a positive GrSR occurred for the abundant phylum of Actinobacteria (*P* < 0.05; Fig. S7), which may be caused by its higher susceptibility to diverse viruses than other phyla [72]. Moreover, we included phylogenetic structure in linear regressions and still found significant (*P* < 0.05) relationships between host growth rate and their interaction specialization *d’* in host-provirus network (Table S4) for abundant phyla such as Actinobacteria (*n* = 745), Delta/epsilon subdivisions (*n* = 60), Firmicutes (*n* = 776) and Proteobacteria (*n* = 1077), which may be contributed by the phylogeny signal of growth rate (supplementary material Note 1). Taken together, these results indicate that GrSRs are negative in some natural environments and specific taxonomies, and thus, in which fast growers are likely to show less specificity to virus clusters than slow growers.

**Fig. 3:**
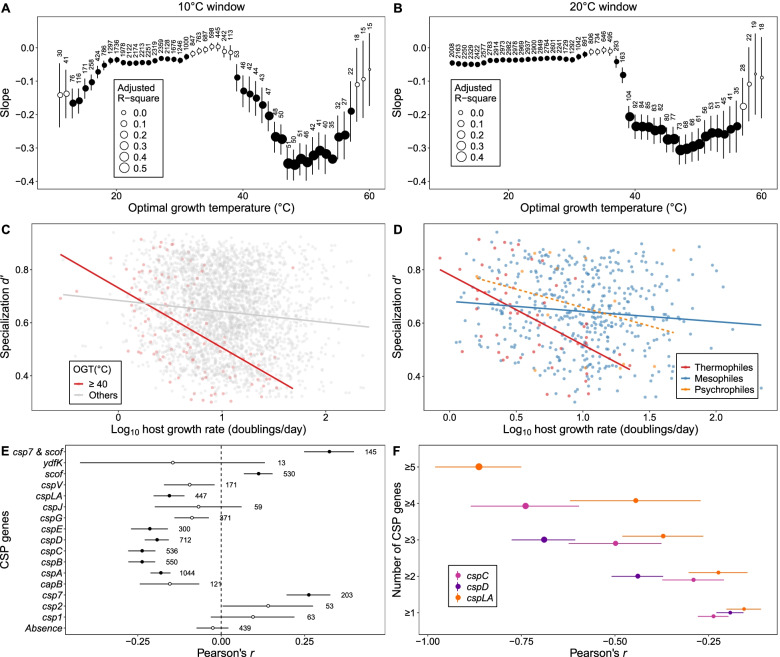
Effects of temperature on GrSRs. The strength of the relationships between host growth rate (log10-scaled) and interaction specialization *d’* was estimated based on 10°C (**A**) and 20°C (**B**) moving windows of the optimal growth temperature (OGT). Linear GrSRs across species OGTs (**C**) and growth temperature ranges (**D**). **E** Pearson’s correlations between host growth rate and *d’* across the presence of various CSP genes. **F** The effects of the number of CSP genes on Pearson’s correlations of the GrSRs. Solid lines denote significant linear relationships (*P* < 0.05), while dashed lines are not linear relationships. Numerical labels represent the number of genomes for analyses. Black points denote statistical significance (*P* < 0.05), while white points indicate nonsignificance. Error lines of points denote standard error

Interestingly, we found that temperature could influence the GrSRs in host-provirus interaction network, which was supported by the following three lines of evidence. First, the slope or strength of negative linear GrSR in terrestrial thermal systems (slope = −0.19 ± 0.07, *P* = 0.01) was generally larger than that in other relatively low-temperature environments (all slopes < 0.12, *P* < 0.05; Figs. 2 and S6). Second, the GrSR strength indicated by the linear slope increased toward high or low OGTs. For example, the result of multiple linear regression analyses showed that growth rate (*P* < 0.001) and OGT (*P* = 0.005) had independent effects on interaction specialization *d’*, while their interaction term was nonsignificant (*P* = 0.803). Moreover, moving window analyses showed that the GrSR rapidly decreased until reaching a plateau phase when OGT gradually increased from 37 to 40°C, (Fig. 3A, B). Consequently, the GrSR strength in host species with OGTs ≥ 40 °C (−0.23 ± 0.04, *P* < 0.001) was stronger than that in other species (−0.04 ± 0.01, *P* < 0.001; Fig. 3C). This temperature range of 37 to 40 °C has been empirically shown to have a wide physiological significance in host-virus interactions by elevating the synthesis of heat shock protein [73] and cell flagellum protein [74], decreasing the activities of the CRISPR-Cas systems in mesophilic bacterium [26] and introducing the lysogeny-to-lysis transition [60]. These physiological statuses are conducive to promoting the infection cycle and thus may strengthen GrSRs. Similarly, the GrSRs gradually decreased when OGT decrease from 20 to 13°C (Fig. 3A). In this temperature range, the expression of cold shock protein [75] and the outer-membrane receptor OmpF was gradually upregulate [25]. Third, the significantly negative GrSRs were stronger in thermophiles (−0.26 ± 0.06, *P* < 0.001; Fig. 3D) than other groups, which was supported by that Deinococcus–Thermus with an OGT range of 50.9 to 66.8 °C have larger GrSR slope (−0.13 ± 0.05, *P* = 0.05; Table S3, Fig. S7) than other phyla, such as Bacteroidetes and Proteobacteria (Fig. S7).

### Molecular mechanisms underlying GrSRs

To mechanistically understand the temperature-dependent GrSRs, we explored potential molecular processes from the perspectives of heat or cold shock responses and the key stages in the infection cycle. Our results showed that heat shock proteins (HSPs) or cold shock proteins (CSPs), protecting cells from lysis damage under high or low temperature stress [76, 77], respectively, potentially mediated the coupling of GrSRs. For HSPs, the presence of their genes (e.g., *hsp40*, *hsp70*, and *hsp100*) resulted in significantly negative GrSRs (Figs. S8 and S9), which were probably contributed by their chaperone activities for lysogeny development [78] and lysis [79].

For CSPs, the absence of their genes showed a non-significant GrSRs (*P* > 0.05; Figs. 3E and S10), while the presence of their genes, such as *csp7*, *cspA*, *cspB*, and *scof*, generally led to significant GrSRs (all *P* < 0.05; Figs. 3E and S10). Furthermore, different CSP genes would cause negative or positive GrSRs. For example, on the one hand, GrSRs were significantly negative for the presence of *cspC*, *cspD*, and *cspLA* (all *P* < 0.05; Figs. 3F and S10), and the higher number of these three CSP genes would result in stronger negative GrSRs (Figs. 3F and S10), which may be associated with the upregulated expression of CSP genes during viral infections [80]. On the other hand, GrSRs were positive for the presence of *csp7* and *scof* genes (*P* < 0.05; Figs. 3E and S10) that were primarily found in Actinobacteria genomes, which implies that both two genes are key genetic traits for the positive GrSR among Actinobacteria. However, at the order level, the abundant groups with *csp7* genes, including Micrococcales, Rhizobiales, Streptomycetales, and Streptosporangiales (all *P* > 0.1; Fig. S11), have no significant GrSR, while Micrococcales with *scof* showed a significantly positive GrSR (*P* < 0.05; Fig. S12). These results imply that the positive GrSR could be taxonomically dependent regarding specific CSP genes, which needs to be further supported by studying more genomes. Overall, we revealed that HSPs and CSPs contribute to the coupling of GrSRs, which may be due to their molecular chaperone activities assisting viral reproduction.

**Fig. 4:**
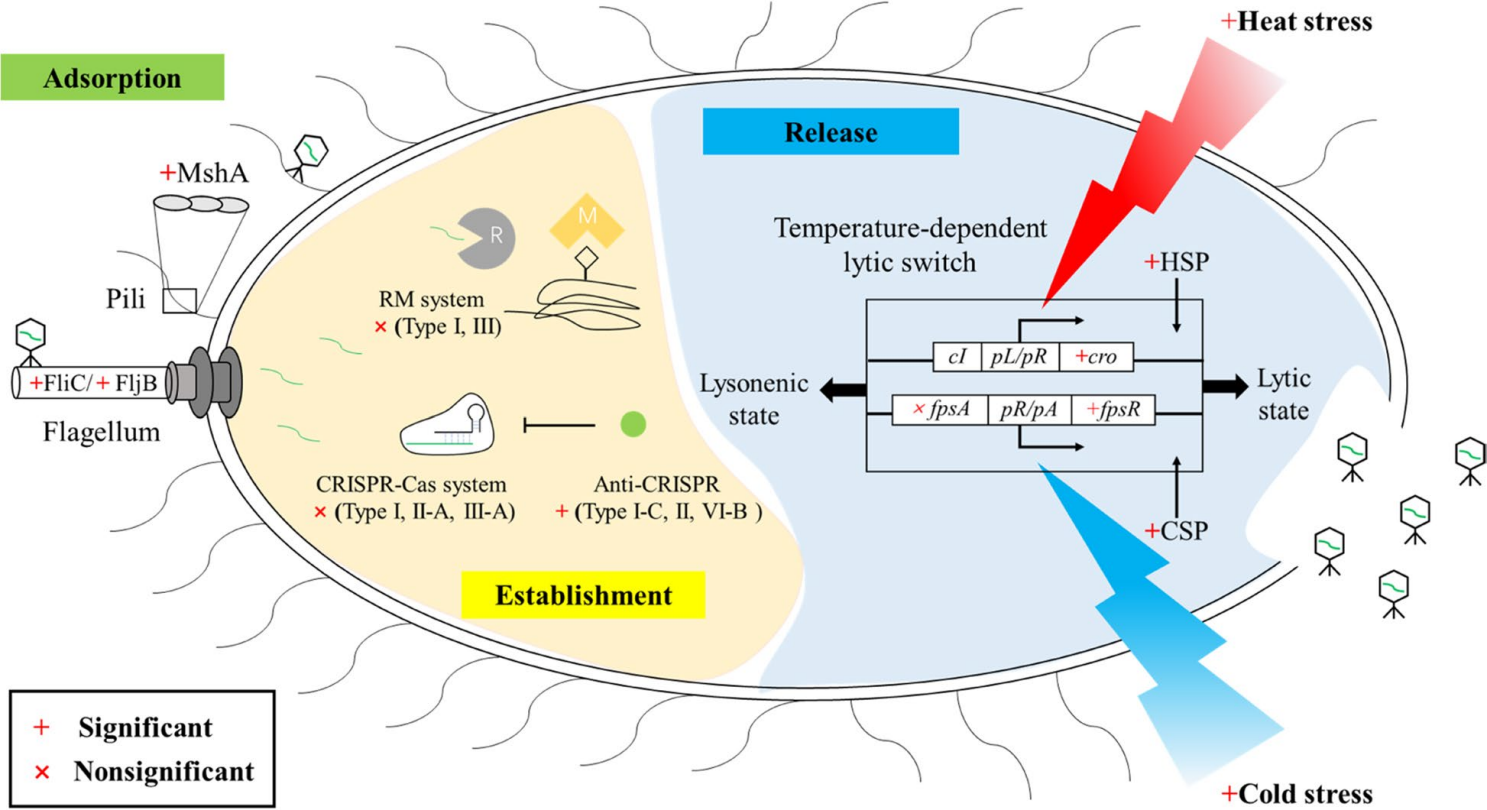
Effects of genes on the negative GrSRs throughout the infection cycle, including the stages of adsorption, establishment, and viral release. The negative GrSRs were significantly (*P* < 0.05) strengthened by the presence of genes responsible for host viral receptors of flagellum and pili, temperature-dependent lytic switches, and phage anti-CRISPR systems but decoupled by host immune systems, including the CRISPR-Cas and RM systems. CRISPR-Cas system: clustered regularly interspaced short palindromic repeats arrays (CRISPR) and CRISPR-associated protein (Cas) system. RM system, restriction-modification system; HSP, heat shock protein; CSP, cold shock protein

Considering that lysis–lysogeny decision of lysogens is obviously influenced by temperature [53, 60], we further investigated how GrSRs are constrained by the genes responsible for the processes in the infection cycle, including key stages of virus adsorption, establishment, and release. For the adsorption stage, host receptors are diverse and play a key role in the specific matching of the host-viral pair [81]. Throughout all receptor proteins, we found that only the presence of genes encoding the flagellum protein FliC or FljB and pilin protein MshA would lead to a significantly negative GrSR (all *P* < 0.05; Fig. 4, Table S5), while this was not the case for outer membrane proteins (*P* > 0.05; Table S5). Compared with the outer membrane proteins, the structural proteins of flagellum and pilus not only serve as viral receptors but also enhance cell motility and form biofilm formation [82] that increase host-virus encounters [16]. For the establishment stage, most of host defense systems generally decoupled GrSRs, including RM systems of types I and III (*P* > 0.05; Figs. 4 and S13) and CRISPR-Cas systems of types I, II, and III (*P* > 0.05; Figs. 4 and S14). This is likely because host defense prevents virus insertion into the host genome and blocks the infection cycle, which is partly supported by the effects of some anti-CRISPR proteins, which resulted in significant GrSRs (all *P* < 0.05; Figs. 4 and S15). For the release stage, considering temperature-dependent GrSRs, we primarily focused on the temperature-dependent lytic switches, such as the CI/Cro repressor [60] and newly identified FpsR [53, 61] for high (≥ 36 °C) and low temperature inductions (≤4 °C), respectively. As expected, the presence of repressor genes, including *cI*/*cro* or *fpsR*, led to a significantly negative GrSR (*P* < 0.05; Figs. 4 and S16), while their absence decoupled GrSRs (*P* > 0.05; Fig. S16). Together, these results highlighted that GrSRs were strengthened by the molecular processes in promoting the infection cycle at the stages of adsorption, establishment, and viral release (*R*^2^_adj_ = 0.182, *P* < 0.001; Fig. S17), but were decoupled by immune systems.

### Mathematical modeling to explain GrSRs

To explore how temperature and population dynamic features modulate the GrSRs, we developed a mathematical model of the population dynamic in host-viral systems using realistic parameters (Fig. S1, Tables S1 and S2) based on the previous frameworks of delay differential equations [57, 58]. Briefly, host-virus systems contained five compartments including susceptible cells, nonsusceptible cells, viruses, infected cell in lysogeny, and lytic state. The transitions between compartments dependent on host-virus infection dynamic in the infection cycle. We assumed that the processes of multiple infection are Bernoulli trails and the induction rate of lysogens is temperature dependent. The temperature thresholds for heat and cold inductions were set as 37°C and 4°C (Fig. S18), respectively. It should be noted that the host growth rate in the modeling system was dependent on the environmental temperature and was not the theoretical maximum value.

**Fig. 5:**
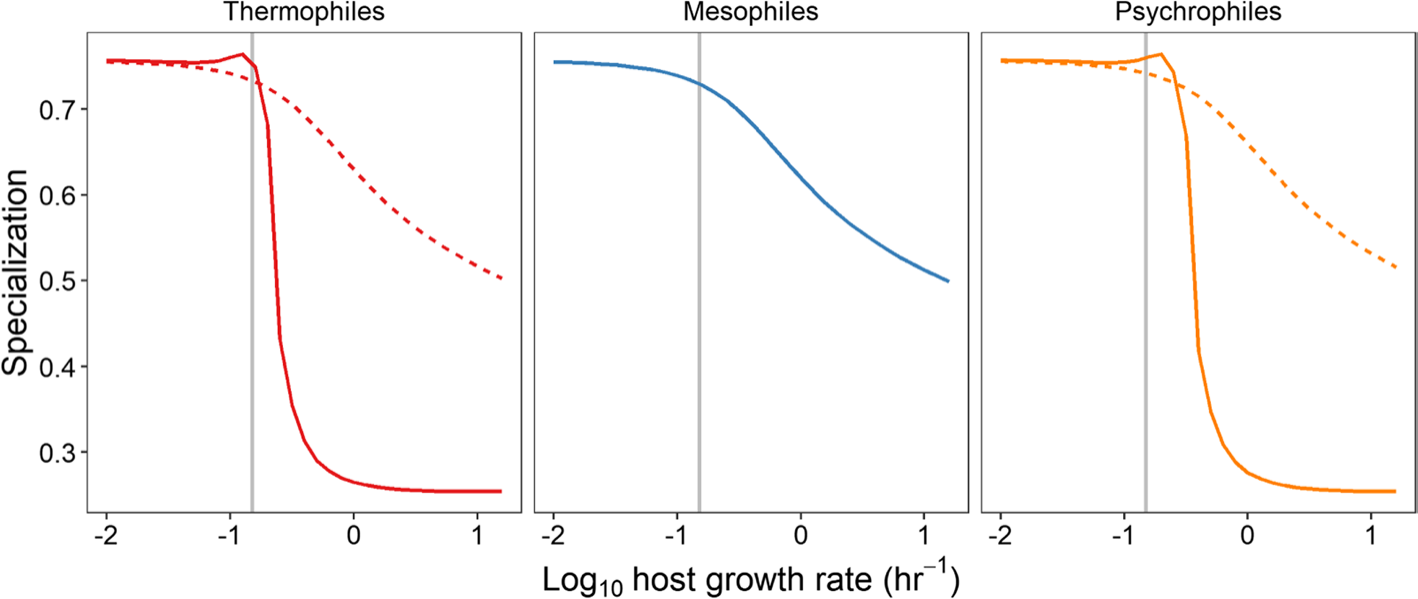
Numerical solutions for the growth rate-specialization relationships (GrSRs) of thermophiles, mesophiles, and psychrophiles. The solid curves represent GrSRs simulated by the mathematical model with a temperature-dependent lytic switch, while dashed curves have no this lytic switch. Thermophiles: OGT = 45°C, environmental temperature = 37°C. Mesophiles: OGT = 30°C, environmental temperature = 30°C. Psychrophiles: OGT = 20°C, environmental temperature = 4°C. Gray vertical lines are the growth rate of nonsusceptible cell population. All parameters used to run the model are listed in Tables S1 and S2

To simplify the questions, interaction specialization of host in modeling system was estimated by the reverse of number of viral species. Our simulation results confirmed the empirical observations showing that thermophiles have a stronger GrSR than mesophiles (Figs. 3D and 5), which may be caused by insufficient virus particles to infect susceptible cells due to the low induction rate at medium temperature. Meanwhile, there was a lag of GrSR curves between thermophiles and psychrophiles toward high host growth rate (Fig. 5), which was consistent with the empirical results that GrSRs in potential psychrophiles harboring CSPs were lagger than those in thermophiles with OGTs ≥ 40°C (Fig. S19). This phenomenon implied that the coupling of GrSRs at low temperature may require a fast growth rate as compensation when the species OGT is greater than the environment temperature of cold induction. Furthermore, GrSR strength were regulated by the temperature-dependent lytic switches. When such lytic switches were removed from the host-virus system, the strengths of GrSR were decreased for thermophiles and psychrophiles (Fig. 5).

### Challenges in testing growth rate-specialization relationships

Based on the solid results of both genomic and modeling analyses, we found that the linear GrSRs are widely existed in Earth’s ecosystems, and its strength is influenced by environment types, host phylogenetic lineages, and traits, which implies that the host-virus interaction specialization at ecosystem levels may be further constrained by environmental factors, such as temperature and nutrient [56]. To confirm and extend these findings, there are at least four challenges to be considered in future studies. First, the GrSR could be further supported by the inclusion of unculturable strains by culture-independent sequencing technology, such as combined use of flow cytometry and single cell sequencing. All lysogenic genomes in this study were culturable isolates compiled from the presently largest dataset [38], which gives an unprecedented scale to investigate the prokaryote-provirus interaction network. Nevertheless, the GrSR also needs an extension to unculturable strains, which may provide novel insights into infection structure due to the unculturable host relying on intercellular metabolic networks [83]. Notably, we have conducted similar analyses for metagenome assembled genomes (MAGs) collected from diverse habitats covering all of Earth’s continents and oceans [84]. Compared to isolate genomes, we found that the results of MAGs were far from reliable due to the following reasons: (1) The reconstruction of MAGs is largely dependent on tetranucleotide frequency, which would leave out many fragments of provirus genome in MAGs. (2) The incompleteness and contamination of MAGs would decrease the prediction accuracy of maximal growth rate and optimal growth temperature. Thus, we expected that metagenome assembled genomes were not suitable for exploring the relationships between host growth rate and their interaction specialization *d’* among unculturable strains.

Second, the host maximal growth rate could be estimated by culture-dependent approaches but it is highly variant even under optimal growth conditions and in the absence of interspecific competition [85]. Our results are mainly based on the maximum host growth rate, which was theoretically predicted by the reverse of the minimal doubling time based on the number of genes encoding ribosomal proteins and the codon usage frequency [85]. In experimental systems, we could use the actual host growth rate in a given environment, like our modeling system, to test whether it has a similar relation to interaction specialization of host during lysogenization. However, we should note that the experimental measured maximum host growth rates may largely dependent on the culture conditions and thus would be different from theoretical maximum host growth rates.

Third, it is challenging to establish and maintain a coculture system containing host species across a large phylogenetic scale. We used a host-provirus network consisting of 3115 host species across 39 phyla to investigate the GrSRs and found that growth rate-specialization relationships were phylogenetically scaled and easier to be found at the higher taxonomic level. For instance, such relationships were significant for 44% of phyla (4 out of 9), 35% of classes (7 out of 12), 19% of orders (10 out of 53), 9% of families (8 out of 88), and 5% of genera (3 out of 56). Thus, GrSR is likely to be found for the groups with common genetic traits rather than specifically taxonomic groups.

Finally, gene manipulations could be used to verify the effects of host genetic traits on GrSRs but it is high cost to develop genetic manipulation system for a large number of species. Alternatively, host strains with a gradient of growth rates belonging to a certain taxonomic group that display GrSRs in genomic analyses could be employed to explore more genes or molecular processes influencing GrSRs. Overcoming the above challenges is key to uncovering the global pattern in the prokaryote-provirus interaction network, which will lead the future researches in host-virus interaction specialization on during host-virus coevolution.

## Conclusions

Exploring the drivers shaping prokaryote-virus network pattern is one of the most important issues in virus ecology. Our study highlights that the prokaryote growth rate play a key role in determining interaction specialization for host in prokaryote-provirus network. The negative growth rate-specialization relationships are widespread in the Earth’s microbiome. Meanwhile, such relationships are temperature-dependent and strengthened by the presence of host genetic traits promoting the infection cycle at the stages of adsorption, establishment, and viral release, but are decoupled by immune systems. Overall, these results help us to uncover the determinants of prokaryote-virus interactions and mechanistically understand their interaction specialization.

## Supplementary Information

**Additional file 1: Table S1.** Model default parameters. **Table S2.** Model initial conditions. **Table S3.** Linear regression model for host growth rate and their virus specificity across different phyla. **Table S4.** Phylogenetic linear regression model for host growth rate and their virus specificity across different phyla. **Table S5.** Effects of the host surface receptors on the GrSRs estimated by linear regression analyses. **Figure S1.** Schematic diagram of modeling host-virus population dynamic. There are five compartments including susceptible cell (*B*^−^, ${B}_0^{-}$), non-susceptible cell (*B*^+^), infected host in lysogeny (${B}_i^{-},i\ne 0$) and lytic state (*L*_*i*_) states, virus (*P*). Arrows denote the transitions between compartments. More details can be saw in the Methods. **Figure S2.** Distribution of the viral cluster number across all species genomes. Such distribution follows the Poisson distribution with an expect value of 1.55. Bar plot are observed values and blue points are predicted values. **Figure S3.** The proportions of proviruses with unavailable genome completeness after the removal of the nodes with low network degrees in the bipartite networks. We considered only the proviruses with unavailable genome completeness for all proviruses or the proviruses of host-provirus networks, and found that the proportion of these proviruses decreased until the network degree ≥ 3. The degrees of 1 and 2 are referring the nodes of singletons and doubletons, respectively. **Figure S4.** The distribution of interaction specialization *d’* for host in the whole host-provirus network (A) and the host-provirus network without singletons and doubletons (B), and the skewness and kurtosis of frequency distribution in the panel B (C) and the panel A (D). Notably, the skewness and kurtosis of frequency distribution indicate the distribution in the panel B is closer to normal distribution than that in the panel A. **Figure S5.** The pairwise correlations among variables. The diagonal plots are the frequency distributions of variables. The plots in upper triangular matrix show the Pearson’s correlation between variables. *d’*: interaction specialization for host. GS: genome size (Mb). GC: GC-content %. CDS: the number of gene coding sequences. 5S rRNA: the number of 5S ribosome RNA. 16S rRNA: the number of 16S ribosome RNA. 23S rRNA: the number of 23S ribosome RNA. 1/NVC: the reverse of number of viral clusters. Gr: host growth rate (doublings/day). OGT: optimal growth temperature (°C). *: *P* < 0.05. **: *P* < 0.01. ***: *P* < 0.001. **Figure S6.** Slopes of the GrSRs across different ecosystems. The slope of GrSR for all environments was estimated by a mixed effect model: *d*’ ~ log_10_(1/DT) + (1 + log_10_(1/DT) | ecosystem). The slope of GrSR for each environment was estimated by linear model: *d*’ ~ log_10_(1/DT). Black points denote significant linear relationships (all *P*_adj_ < 0.05), while white points are nonsignificant. Point size represents adjust R-square. **Figure S7.** The GrSRs across phyla. Solid lines are significant linear relationships (*P*_adj_ < 0.05), while dashed lines are nonsignificant. **Figure S8.** Effects of the genes encoding heat shock proteins (HSP) on GrSRs. Solid lines denote significant linear relationships (*P*_adj_ < 0.05), while dashed lines are nonsignificant. **Figure S9.** The GrSR slopes across the number of genes encoding heat shock proteins (HSP). Numerical labels represent the number of genomes for analyses. Black points denote significant linear relationships (*P*_adj_ < 0.05), while grey points are nonsignificant. Error lines denote standard error. **Figure S10.** Effects of the genes encoding cold shock proteins (CSP) on GrSRs. Solid lines denote significant linear relationships (*P*_adj_ < 0.05), while dashed lines are nonsignificant. **Figure S11.** Effects of the *csp7* genes on GrSRs across main orders. Dashed lines denote nonsignificant linear relationships (all *P* > 0.1). **Figure S12.** Effects of the *scoF* genes on GrSRs across various orders. Solid lines denote significant linear relationships (*P*_adj_ < 0.05), while dashed lines are nonsignificant. **Figure S13.** Effects of the RM systems on GrSRs. Solid lines denote significant linear relationships (*P*_adj_ < 0.05), while dashed lines are nonsignificant. **Figure S14.** Effects of the CRISPR-Cas systems on GrSRs. Solid lines denote significant linear relationships (*P*_adj_ < 0.05), while dashed lines are nonsignificant. **Figure S15.** Effects of the anti-CRISPR systems on GrSRs. Solid lines denote significant linear relationships (*P*_adj_ < 0.05), while dashed lines are nonsignificant. **Figure S16.** Effects of the temperature-dependent lytic switches on GrSRs. Solid lines denote significant linear relationships (*P*_adj_ < 0.05), while dashed lines are nonsignificant. **Figure S17.** GrSRs for those species harboring the genes involving in the molecular processes in promoting the infection cycle at the stages of adsorption, establishment and viral release (*R*^2^_adj_ = 0.182, *P* < 0.001, df = 154). Those species harbor at least one gene encoding heat shock protein (*hsp20*, *hsp40*, *hsp70* or *hsp100*), at least two genes encoding cold shock protein (*cspC*, *cspD*, or *cspLA*), at least one gene encoding receptor protein (*fliC*, *fljB* or *mshA*), at least one pair of genes encoding heat lytic switch (*cI* and *cro*) and at least one gene encoding cold lytic switch (*fpsR*). **Figure S18.** Temperature-dependent induction. It is controlled by two lytic switches including the repressors of CI/Cro (for high temperature at 37°C) and FpsR (for low temperature at 4 °C), which is simulated by a Sigmond function. **Figure S19.** Significant linear GrSRs of the potential psychrophiles harboring at least three CSP genes and the thermophiles with OGT larger than 40°C. All *P*_adj_ < 0.05.

## Data Availability

The codes and datasets generated during the current study are available in the online repository, https://github.com/zhenghualiu/GrSRs.
