# Supplementary material for: Growth rate determines prokaryote-provirus network modulated by temperature and host genetic traits
Source: Microbiome. 2022 Jun 14;10:92. doi: 10.1186/s40168-022-01288-x (PMC9195381; doi:10.1186/s40168-022-01288-x)
Supplement: Supplementary file 2 — Additional file 1: Table S1. Model default parameters. Table S2. Model initial conditions. Table S3. Linear regression model for host growth rate and their virus specificity across different phyla. Table S4. Phylogenetic linear regression model for host growth rate and their virus specificity across different phyla. Table S5. Effects of the host surface receptors on the GrSRs estimated by linear regression analyses. Figure S1. Schematic diagram of modeling host-virus population dynamic. There are five compartments including susceptible cell (B−, ${B}_0^{-}$B0-), non-susceptible cell (B+), infected host in lysogeny (${B}_i^{-},i\ne 0$Bi-,i≠0) and lytic state (Li) states, virus (P). Arrows denote the transitions between compartments. More details can be saw in the Methods. Figure S2. Distribution of the viral cluster number across all species genomes. Such distribution follows the Poisson distribution with an expect value of 1.55. Bar plot are observed values and blue points are predicted values. Figure S3. The proportions of proviruses with unavailable genome completeness after the removal of the nodes with low network degrees in the bipartite networks. We considered only the proviruses with unavailable genome completeness for all proviruses or the proviruses of host-provirus networks, and found that the proportion of these proviruses decreased until the network degree ≥ 3. The degrees of 1 and 2 are referring the nodes of singletons and doubletons, respectively. Figure S4. The distributi [file 40168_2022_1288_MOESM1_ESM.docx]

Supplementary Information for

Growth rate determines prokaryote-provirus network modulated by temperature and host genetic traits

Zhenghua Liu^1,2^, Qingyun Yan^3^, Chengying Jiang^4^, Juan Li^5^, Huahua Jian^6^, Lu Fan^7^, Rui Zhang^8^, Xiang Xiao^6^, Delong Meng^1^, Xueduan Liu^1^, Jianjun Wang^2*^, Huaqun Yin^1*^

**The file includes:**

Supplementary Tables (S1–S5)

Supplementary Figures (S1–S19)

**Table S1.** Model default parameters

| Parameter | Description | Default value | Reference |
| --- | --- | --- | --- |
| *C* | Environment capacity | 2·10^8^ cells/mL | ~ 10^8^ cells/mL (Pleska et al., 2018) |
| $\alpha$ | Probability of lysogeny | 0.5 | - |
| $\sigma_{+}$ , $\sigma_{-}$ | Niche breadth of temperature | 15 K | - |
| $\delta_{0}$ | Adsorption rate constant | 4·10^-8^ mL/(hr·K^1/2^) | 2.3·10^-9^ mL/(hr·K^1/2^) (Pleska et al., 2018) |
| $\lambda_{0}$ | Basic number of virus species infecting cell | 1.5 particles | - |
| $\tau$ | Latent period | 1 h | 1 h (Pleska et al., 2018) |
| $\xi_{0}$ | Induction rate constant | 2·10^-5^ hr^-1^ | 2.4·10^-5^ hr^-1^ (Pleska et al., 2018) |
| $\beta_{0}$ | Basic replication factor | 5·10^2^ | (1.5±0.5)·10^2^ (Egilmez et al., 2018) |
| $T_{h}$ | Temperature of heat-activated switch | 273.15+37 K | 308.15-310.15 K (Bednarz et al., 2014) |
| $T_{c}$ | Temperature of cold-activated switch | 273.15+4 K | 277.15 K (Meng et al., 2020) |
| $k$ | Constant of switch function | 4 | - |
| $T_{0}$ | Optimal growth temperature of thermophiles | 273.15+45 K | 318.15-343.15 K |
|  | Optimal growth temperature of mesophiles | 273.15+30 K | 293.15-318.15 K |
|  | Optimal growth temperature of psychrophiles | 273.15+20 K | ＞273.15+15 K (Moyer and Morita, 2007) |
| $V_{+}$ | Maximal growth rate of perfect immune cell | 0.15 hr^-1^ | - |

**Table S2.** Model initial conditions

| State | Description | Initial condition |
| --- | --- | --- |
| $B^{-}$ | Population density of sensitive cell | 4·10^6^ cells/mL |
| $B_{i}^{-}$ | Population density of lysogeny | 0 |
| $P$ | Virus density | 1·10^5^ particles/mL |

**Table S3.** Linear regression model for host growth rate and their virus specificity across different phyla.

| Phylum | n | Term | Estimate | StdErr | *t* | *P*_adj_ | R^2^_adj_ | RMSE | MAE | Centre of residuals |
| --- | --- | --- | --- | --- | --- | --- | --- | --- | --- | --- |
| **All** | **3065** | **log_10_(1/DT)** | **-0.0430** | **0.0064** | **-6.7755** | **0.0000** | **0.0144** | **0.1447** | **0.1223** | **-1.62E-17** |
| Acidobacteria | 9 | log_10_(1/DT) | 0.0820 | 0.0461 | 1.7768 | 0.1430 | 0.2124 | 0.0274 | 0.0207 | 1.15E-18 |
| **Actinobacteria** | **745** | **log_10_(1/DT)** | **0.0528** | **0.0172** | **3.0778** | **0.0110** | **0.0113** | **0.1359** | **0.1127** | **2.23E-18** |
| **Bacteroidetes** | **259** | **log_10_(1/DT)** | **-0.0528** | **0.0214** | **-2.4625** | **0.0363** | **0.0192** | **0.1153** | **0.1007** | **3.81E-19** |
| Cyanobacteria | 17 | log_10_(1/DT) | -0.0416 | 0.2003 | -0.2077 | 0.8383 | NA | 0.0802 | 0.0634 | -7.83E-18 |
| **Deinococcus-Thermus** | **9** | **log_10_(1/DT)** | **-0.1273** | **0.0449** | **-2.8338** | **0.0500** | **0.4678** | **0.0301** | **0.0246** | **-1.44E-19** |
| Delta/epsilon subdivisions | 60 | log_10_(1/DT) | 0.0891 | 0.0559 | 1.5942 | 0.1430 | 0.0255 | 0.1382 | 0.1107 | -7.66E-19 |
| Euryarchaeota | 67 | log_10_(1/DT) | 0.0912 | 0.0478 | 1.9092 | 0.1010 | 0.0385 | 0.0814 | 0.0651 | -1.41E-18 |
| Firmicutes | 776 | log_10_(1/DT) | -0.0176 | 0.0116 | -1.5210 | 0.1430 | 0.0017 | 0.1474 | 0.1248 | 4.83E-18 |
| **Proteobacteria** | **1077** | **log_10_(1/DT)** | **-0.0288** | **0.0101** | **-2.8589** | **0.0143** | **0.0066** | **0.1354** | **0.1135** | **-7.12E-18** |

StdErr: standard error. RMSE: Root mean square error. MAE: Mean absolute error.

P values are adjusted by Benjamini-Hochberg method.

**Table S4.** Phylogenetic linear regression model for host growth rate and their virus specificity across different phyla.

| Phylum | Model | n | Term | Estimate | StdErr | *t* | lowerbootCI | upperbootCI | *P* | R^2^_adj_ | RMSE | MAE | Centre of residuals |
| --- | --- | --- | --- | --- | --- | --- | --- | --- | --- | --- | --- | --- | --- |
| All | BM | 3065 | log_10_(1/DT) | **1.4536** | **0.0440** | **33.0272** | **1.3969** | **1.5371** | **0.0000** | **0.2624** | **1.1695** | **1.0010** | **-0.9834** |
|  | delta |  |  | **1.2742** | **0.0500** | **25.6781** | **1.2111** | **1.3651** | **0.0000** | **0.1769** | **1.0339** | **0.8923** | **-0.8683** |
| Acidobacteria | BM | 9 | log_10_(1/DT) | 0.0993 | 0.0589 | 1.6842 | 0.0078 | 0.1859 | 0.1360 | 0.1867 | 0.0281 | 0.0218 | -0.0048 |
|  | delta |  |  | 0.1016 | 0.0539 | 1.8835 | 0.0231 | 0.1829 | 0.1016 | 0.2415 | 0.0282 | 0.0219 | -0.0047 |
| Actinobacteria | BM | 745 | log_10_(1/DT) | **1.1634** | **0.0899** | **12.9420** | **0.9875** | **1.3413** | **0.0000** | **0.1829** | **0.8064** | **0.7327** | **-0.7264** |
|  | delta |  |  | 0.1080 | 0.0978 | 1.1043 | -0.0506 | 0.2466 | 0.2698 | 0.0003 | **0.1668** | **0.1377** | **-0.0953** |
| Bacteroidetes | BM | 259 | log_10_(1/DT) | -0.0371 | 0.0411 | -0.9031 | -0.0934 | 0.0191 | 0.3673 | NA | 0.1163 | 0.1001 | 0.0140 |
|  | delta |  |  | -0.0436 | 0.0395 | -1.1043 | -0.1216 | 0.0380 | 0.2705 | 0.0008 | 0.1161 | 0.1008 | 0.0128 |
| Cyanobacteria | BM | 17 | log_10_(1/DT) | 1.2267 | 1.1145 | 1.1007 | -0.9622 | 3.1340 | 0.2884 | NA | 0.2022 | 0.1764 | 0.1314 |
|  | delta |  |  | 1.3361 | 1.1491 | 1.1628 | -0.9005 | 3.5594 | 0.2631 | 0.0215 | 0.2330 | 0.2078 | 0.1660 |
| Deinococcus-Thermus | BM | 9 | log_10_(1/DT) | -0.0885 | 0.1087 | -0.8145 | -0.3104 | 0.0993 | 0.4422 | NA | 0.0328 | 0.0279 | -0.0085 |
|  | delta |  |  | -0.1015 | 0.0824 | -1.2313 | -0.2379 | 0.0363 | 0.2580 | 0.0606 | 0.0314 | 0.0269 | -0.0061 |
| Delta/epsilon subdivisions | BM | 60 | log_10_(1/DT) | **-0.1865** | **0.0859** | **-2.1713** | **-0.3632** | **0.0170** | **0.0340** | **0.0592** | **0.1696** | **0.1442** | **0.0408** |
|  | delta |  |  | -0.1366 | 0.0858 | -1.5927 | -0.3046 | 0.0488 | 0.1167 | 0.0234 | 0.1635 | 0.1400 | 0.0479 |
| Euryarchaeota | BM | 67 | log_10_(1/DT) | 0.1079 | 0.0880 | 1.2260 | -0.0398 | 0.2914 | 0.2246 | 0.0076 | 0.0880 | 0.0750 | 0.0334 |
|  | delta |  |  | 0.1265 | 0.0877 | 1.4429 | -0.1092 | 0.2555 | 0.1539 | 0.0161 | 0.0861 | 0.0723 | 0.0272 |
| Firmicutes | BM | 776 | log_10_(1/DT) | **3.5811** | **0.0129** | **277.2652** | **3.5556** | **3.5995** | **0.0000** | **0.9900** | **2.4168** | **2.0521** | **-1.7646** |
|  | delta |  |  | **3.5796** | **0.0132** | **271.7147** | **3.5489** | **3.6103** | **0.0000** | **0.9896** | 2.4589 | 2.0910 | -1.8224 |
| Proteobacteria | BM | 1077 | log_10_(1/DT) | **3.2881** | **0.0531** | **61.9012** | **3.1826** | **3.3824** | **0.0000** | **0.7807** | **1.6720** | **1.3444** | **-0.9643** |
|  | delta |  |  | **3.3272** | **0.0522** | **63.7699** | **3.1970** | **3.4092** | **0.0000** | **0.7907** | 1.8286 | 1.4836 | -1.1976 |

StdErr: standard error. RMSE: Root mean square error. MAE: Mean absolute error.

**Note 1:** Before applying a phylogenetic correction, we found that in linear model $d^{'}=\beta*X+ \varepsilon(X={log}_{10}(1/DT))$, the error term $\varepsilon$ has extremely weak phylogenetic signal (*K* = 0.000007, *P* = 0.074) although the species trait of growth rate $X$ has significant phylogenetic signal (*K* = 0.0017, *P* = 0.001). In this case, ordinary least squares yielded a better parameter estimate of the slope $\beta$ than phylogenetic generalized least squares (Revell, 2010), which is supported by the lower values of root mean square error (RMSE) and the mean absolute error (MAE) of linear model than phylogenetic linear model (Table S3 and S4). We also noted that the centres of residuals were closed to be zero for linear models but not for phylogenetic linear models (Table S4), which indicates that the estimates of phylogenetic linear models were overestimated or underestimated, and thus caused the switching direction. Taken together, we considered that the effects of phylogeny on significant slope $\beta$ are primarily contributed by species traits $X$ rather than the error term $\varepsilon$.

Phylogenetic signals of $X$ and $\varepsilon$ were computed by phylosig function via K method within ‘phytools’ 1.0-3 package (Revell, 2012).

**Table S5.** Effects of the host surface receptors on the GrSRs estimated by linear regression analyses.

| Gene | Receptor | Slope | N | SD | R^2^_adj_ | *P*_adj_ |
| --- | --- | --- | --- | --- | --- | --- |
| **Absence** | **NA** | **-0.032** | **1836** | **0.009** | **0.007** | **0.0086** |
| BactA | Probable bacteriophage receptor BactA | -0.317 | 5 | 0.227 | 0.193 | 0.6933 |
| BtuB | Vitamin B12 transporter BtuB | 0.012 | 128 | 0.041 | -0.007 | 0.8634 |
| ECs0179 | Outer membrane protein assembly factor BamA | 0.025 | 203 | 0.030 | -0.001 | 0.6933 |
| ECs3104 | Outer membrane porin C | 0.069 | 8 | 0.048 | 0.134 | 0.6933 |
| FadL | Long-chain fatty acid transport protein | 0.019 | 134 | 0.039 | -0.006 | 0.8229 |
| FepA | Outer membrane porin, receptor for ferric enterobactin (Enterochelin) and colicins B and D | -0.015 | 150 | 0.022 | -0.004 | 0.7689 |
| FhuA | Ferrichrome outer membrane transporter/phage receptor | 0.007 | 82 | 0.039 | -0.012 | 0.9068 |
| **FliC** | **Flagellin** | **-0.065** | **292** | **0.019** | **0.037** | **0.0086** |
| FliK | Flagellar hook-length control protein | 0.078 | 44 | 0.055 | 0.022 | 0.6933 |
| **FljB** | **Phase 2 flagellin** | **-0.050** | **450** | **0.016** | **0.020** | **0.0112** |
| LamB | Maltoporin | 0.034 | 123 | 0.043 | -0.003 | 0.6933 |
| **MshA** | **18.1 kDa type 4 prepilin, structural subunit of mannoses. Pili** | **-0.042** | **578** | **0.013** | **0.017** | **0.0110** |
| NfrA | Bacteriophage adsorption protein A | 0.028 | 4 | 0.372 | -0.496 | 0.9478 |
| OmpA | Outer membrane protein A | 0.006 | 249 | 0.021 | -0.004 | 0.8634 |
| OmpC | Outer membrane porin C | 0.038 | 91 | 0.043 | -0.002 | 0.6933 |
| OmpF | Outer membrane porin F | 0.020 | 136 | 0.039 | -0.006 | 0.8229 |
| OmpLC | Outer membrane porin protein LC | 0.000 | 2 | NA | NA | NA |
| PhoE | Outer membrane porin PhoE | 0.038 | 79 | 0.045 | -0.004 | 0.6933 |
| Pip | Phage infection protein | 0.092 | 27 | 0.102 | -0.007 | 0.6933 |
| Prn | Pertactin | -0.445 | 2 | NA | NA | NA |
| PSARA30_16 | Type IV protein secretion/mating pair formation complex protein | -0.131 | 12 | 0.100 | 0.061 | 0.6933 |
| Sap | S-layer protein sap | -0.293 | 2 | NA | NA | NA |
| TolA | Tol-Pal system protein TolA | 0.016 | 133 | 0.040 | -0.006 | 0.8400 |
| TolC | Outer membrane protein TolC | 0.026 | 199 | 0.030 | -0.001 | 0.6933 |
| TonB | Protein TonB | 0.017 | 139 | 0.037 | -0.006 | 0.8229 |
| Tsx | Nucleoside-specific channel-forming protein Tsx | 0.002 | 124 | 0.036 | -0.008 | 0.9478 |
| VAA_RS04305 | Outer membrane protein ompK | 0.103 | 47 | 0.063 | 0.035 | 0.5824 |
| VAS14_16696 | Outer membrane protein OmpK | -0.177 | 17 | 0.159 | 0.014 | 0.6933 |
| VC0633 | Outer membrane protein U | 0.447 | 11 | 0.321 | 0.085 | 0.6933 |
| VC1837 | TolA protein | 0.160 | 19 | 0.200 | -0.020 | 0.6933 |
| VCA0867 | Outer membrane protein W | 0.053 | 97 | 0.048 | 0.003 | 0.6933 |
| YjaE | Uncharacterized protein | 0.109 | 35 | 0.051 | 0.096 | 0.2521 |
| YncD | Probable TonB-dependent receptor YncD | 0.019 | 92 | 0.041 | -0.009 | 0.8229 |
| YueB | ESX secretion system protein YueB | 0.021 | 10 | 0.068 | -0.111 | 0.8634 |

N: Number of observation points. SD: Standard deviation. *P*_adj_: *P* value adjusted by Benjamini-Hochberg method.


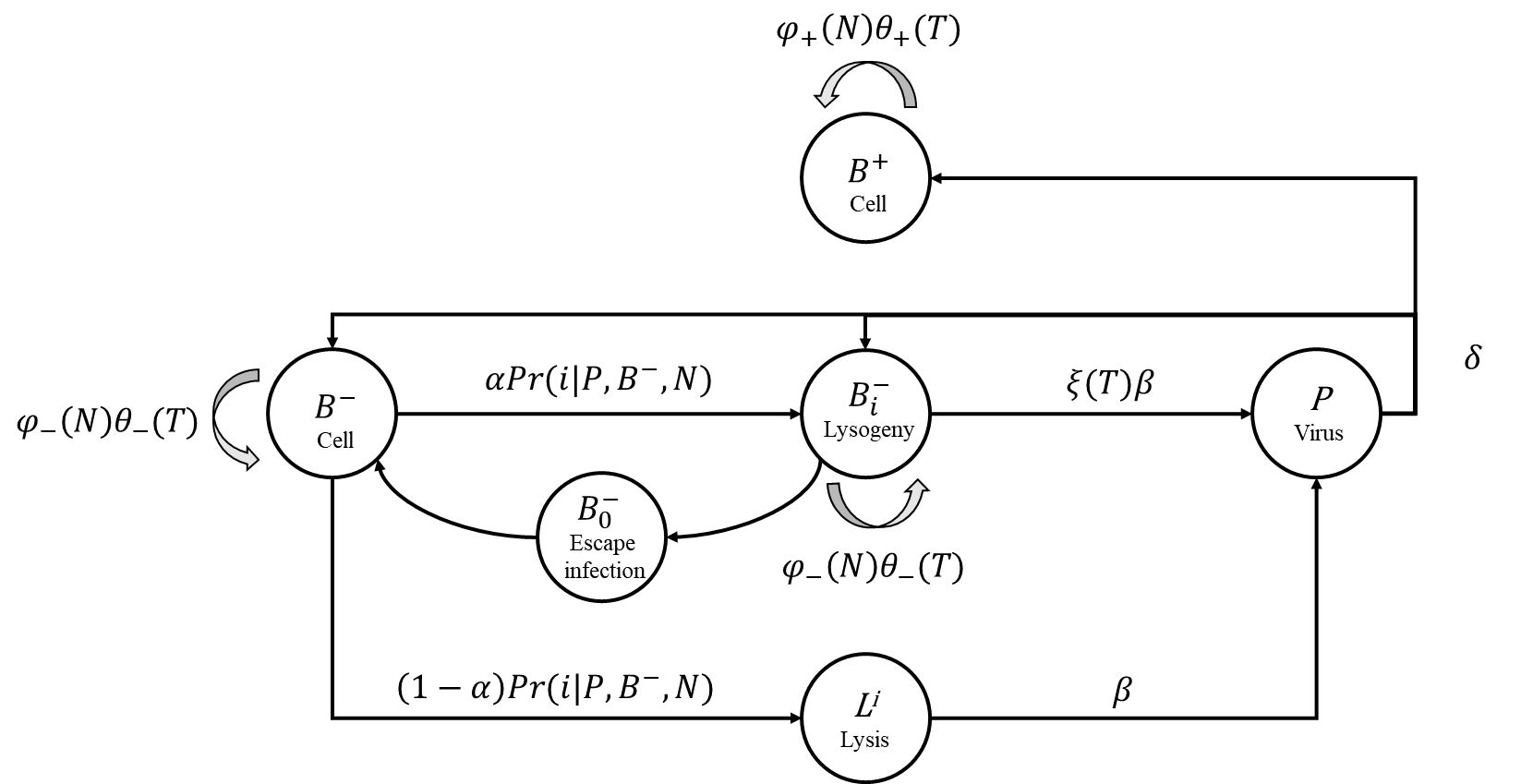


**Figure S1.** Schematic diagram of modeling host-virus population dynamic. There are five compartments including susceptible cell ($B^{-}$, $B_{0}^{-}$), non-susceptible cell ($B^{+}$), infected host in lysogeny ($B_{i}^{-}, i\neq0$) and lytic state ($L_{i}$) states, virus (*P*). Arrows denote the transitions between compartments. More details can be saw in the Methods.


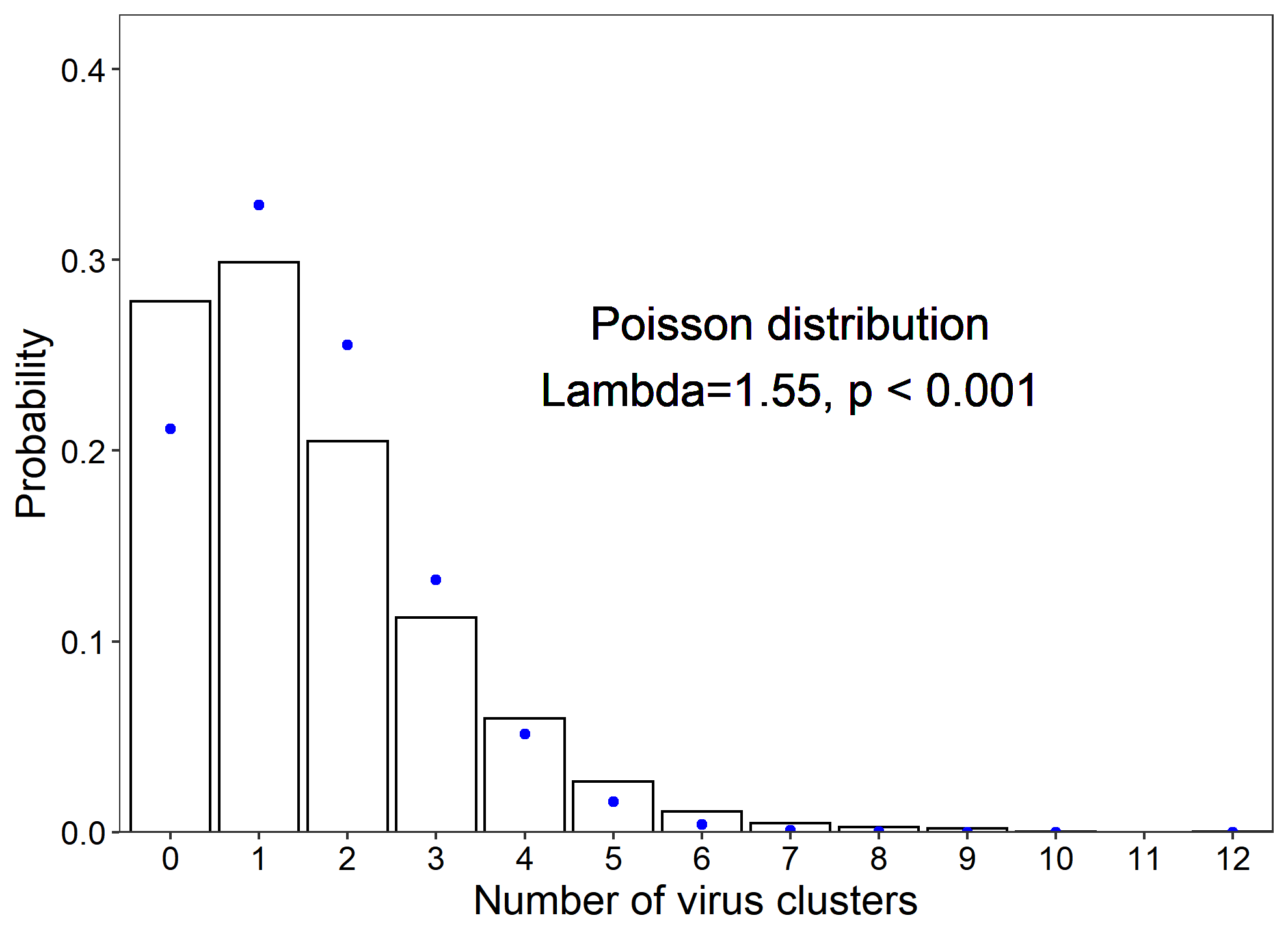


**Figure S2.** Distribution of the viral cluster number across all species genomes. Such distribution follows the Poisson distribution with an expect value of 1.55. Bar plot are observed values and blue points are predicted values.


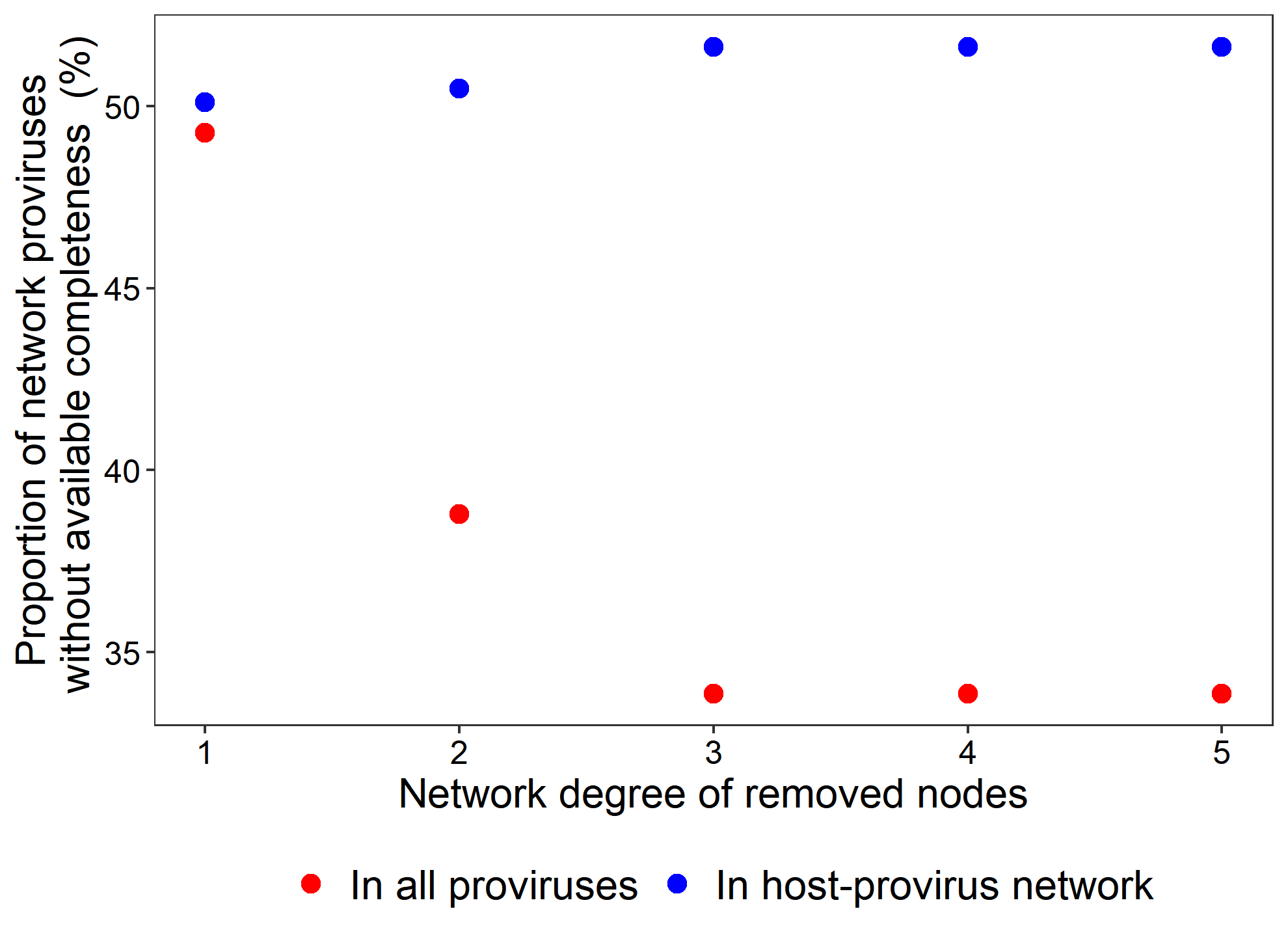


**Figure S3.** The proportions of proviruses with unavailable genome completeness after the removal of the nodes with low network degrees in the bipartite networks. We considered only the proviruses with unavailable genome completeness for all proviruses or the proviruses of host-provirus networks, and found that the proportion of these proviruses decreased until the network degree ≥ 3. The degrees of 1 and 2 are referring the nodes of singletons and doubletons, respectively.


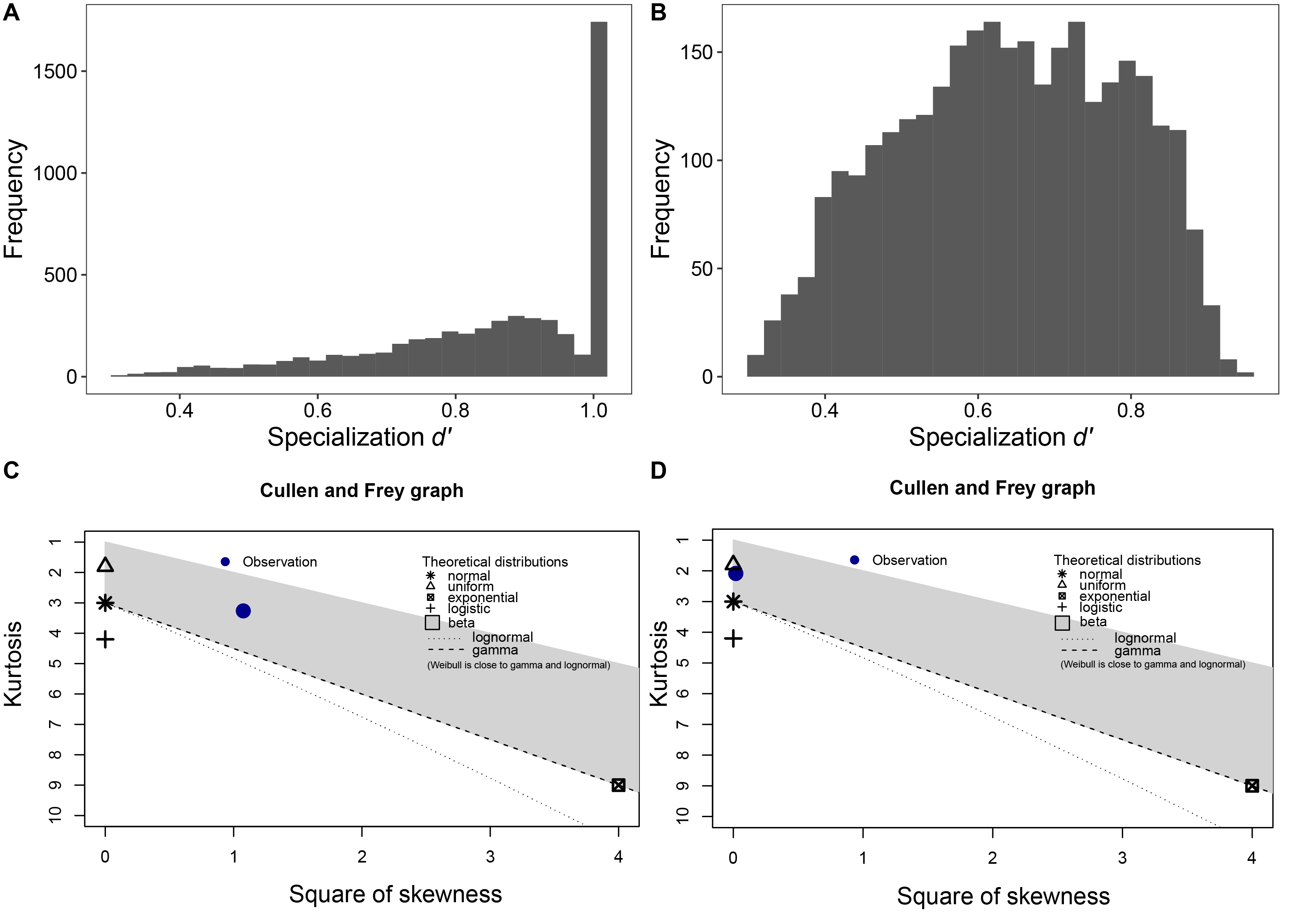


**Figure S4.** The distribution of interaction specialization *d’* for host in the whole host-provirus network (A) and the host-provirus network without singletons and doubletons (B), and the skewness and kurtosis of frequency distribution in the panel B (C) and the panel A (D). Notably, the skewness and kurtosis of frequency distribution indicate the distribution in the panel B is closer to normal distribution than that in the panel A.


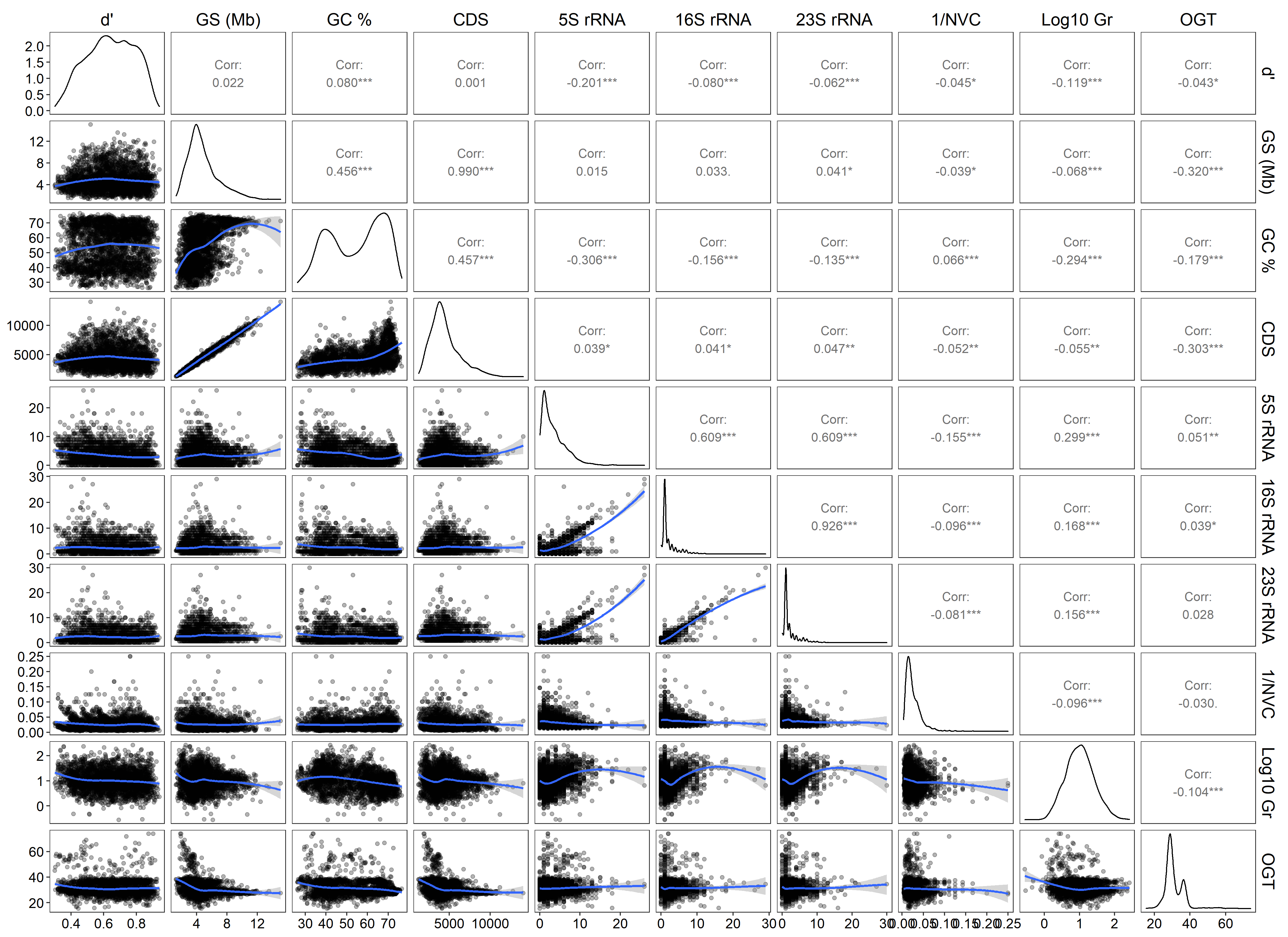


**Figure S5.** The pairwise correlations among variables. The diagonal plots are the frequency distributions of variables. The plots in upper triangular matrix show the Pearson’s correlation between variables. *d'*: interaction specialization for host. GS: genome size (Mb). GC: GC-content %. CDS: the number of gene coding sequences. 5S rRNA: the number of 5S ribosome RNA. 16S rRNA: the number of 16S ribosome RNA. 23S rRNA: the number of 23S ribosome RNA. 1/NVC: the reverse of number of viral clusters. Gr: host growth rate (doublings/day). OGT: optimal growth temperature (℃). *: *P* < 0.05. **: *P* < 0.01. ***: *P* < 0.001.


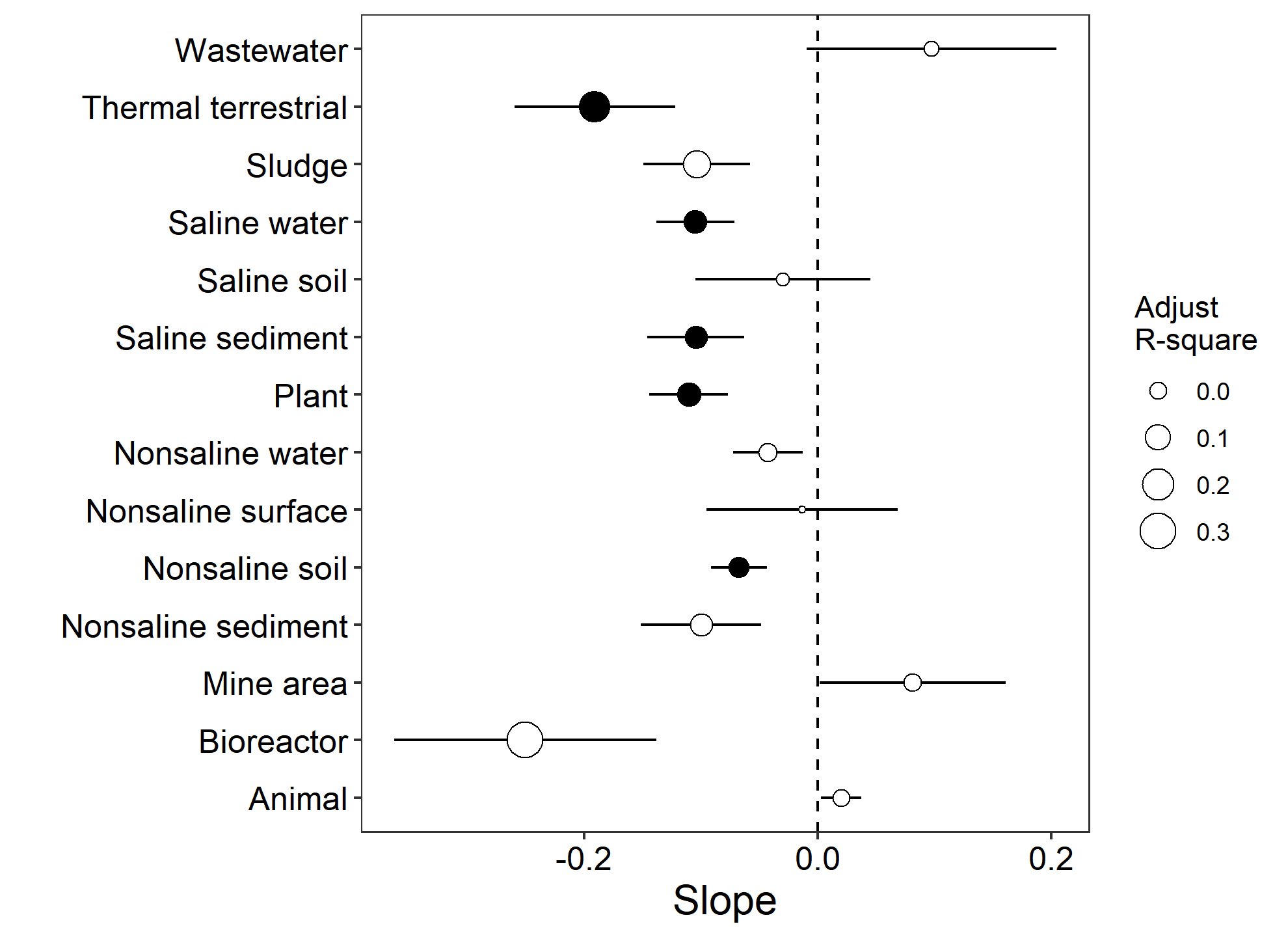


**Figure S6. Slopes of the GrSRs across different** **ecosystems.** The slope of GrSR for all environments was estimated by a mixed effect model: *d*’ ~ log_10_(1/DT) + (1 + log_10_(1/DT) | ecosystem). The slope of GrSR for each environment was estimated by linear model: *d*’ ~ log_10_(1/DT). Black points denote significant linear relationships (all *P*_adj_ < 0.05), while white points are nonsignificant. Point size represents adjust R-square.


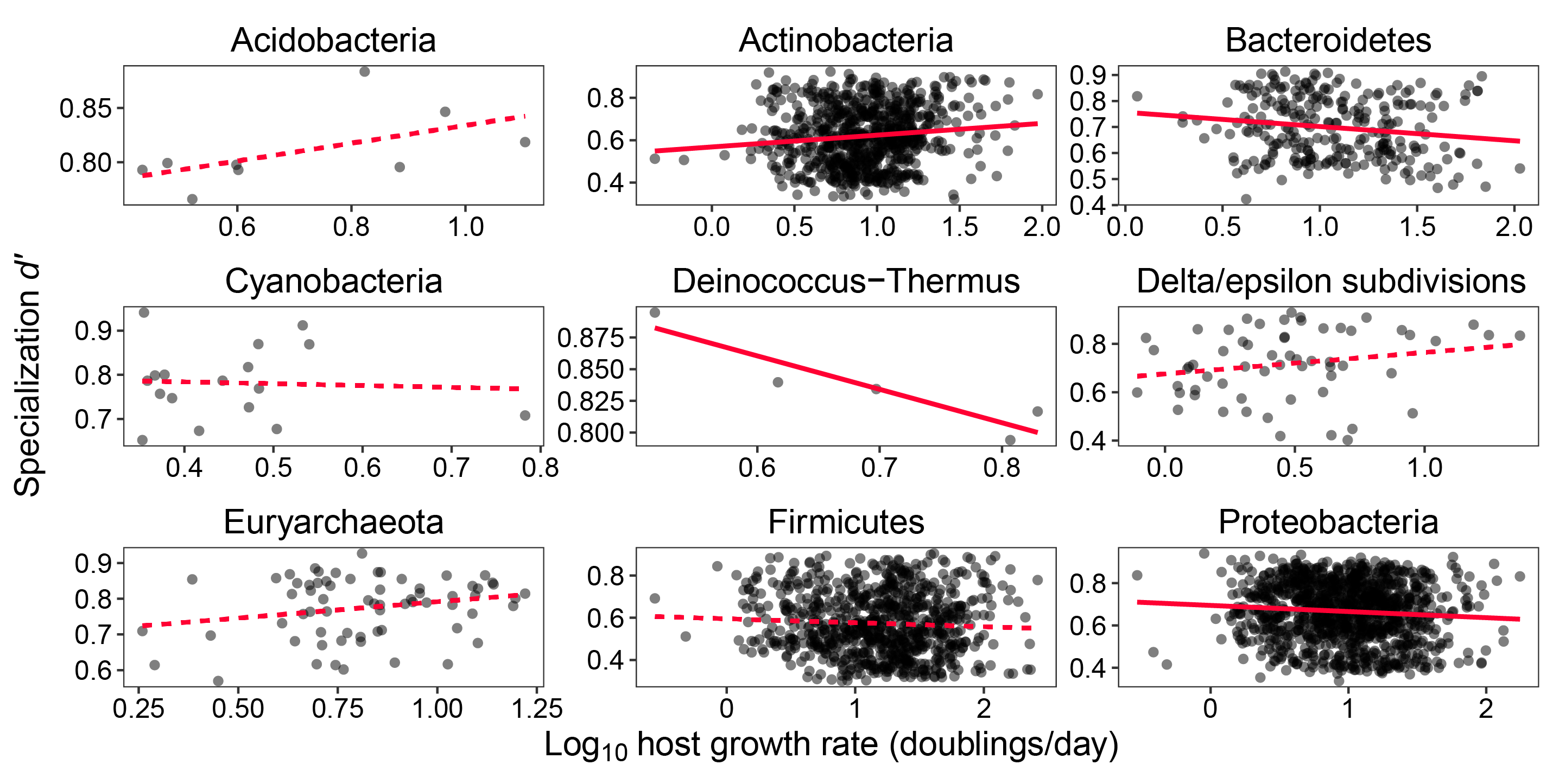


**Figure S7. The GrSRs across phyla.** Solid lines are significant linear relationships (*P*_adj_ < 0.05), while dashed lines are nonsignificant.


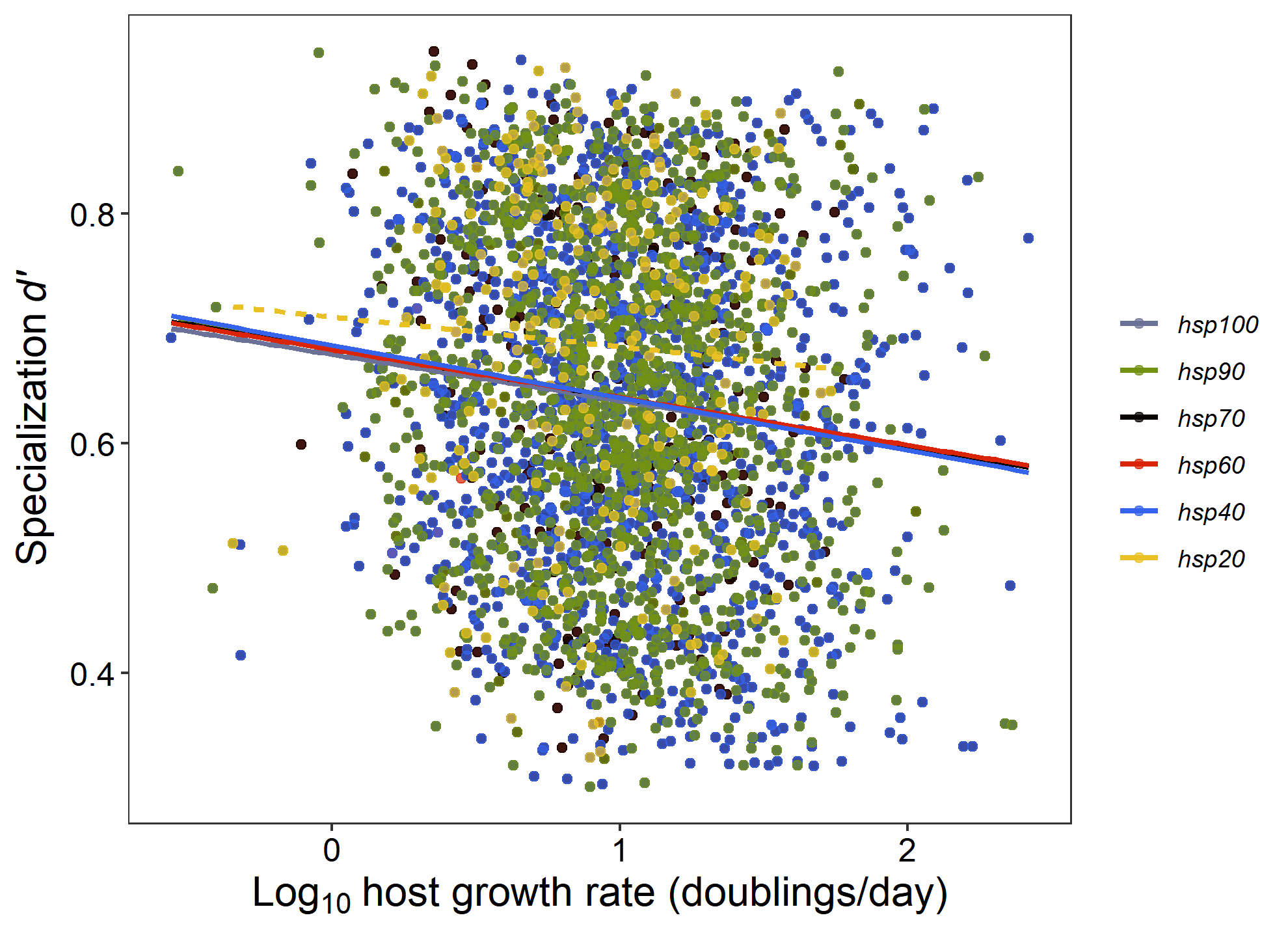


**Figure S8. Effects of the genes encoding heat shock proteins (HSP) on GrSRs.** Solid lines denote significant linear relationships (*P*_adj_ < 0.05), while dashed lines are nonsignificant.


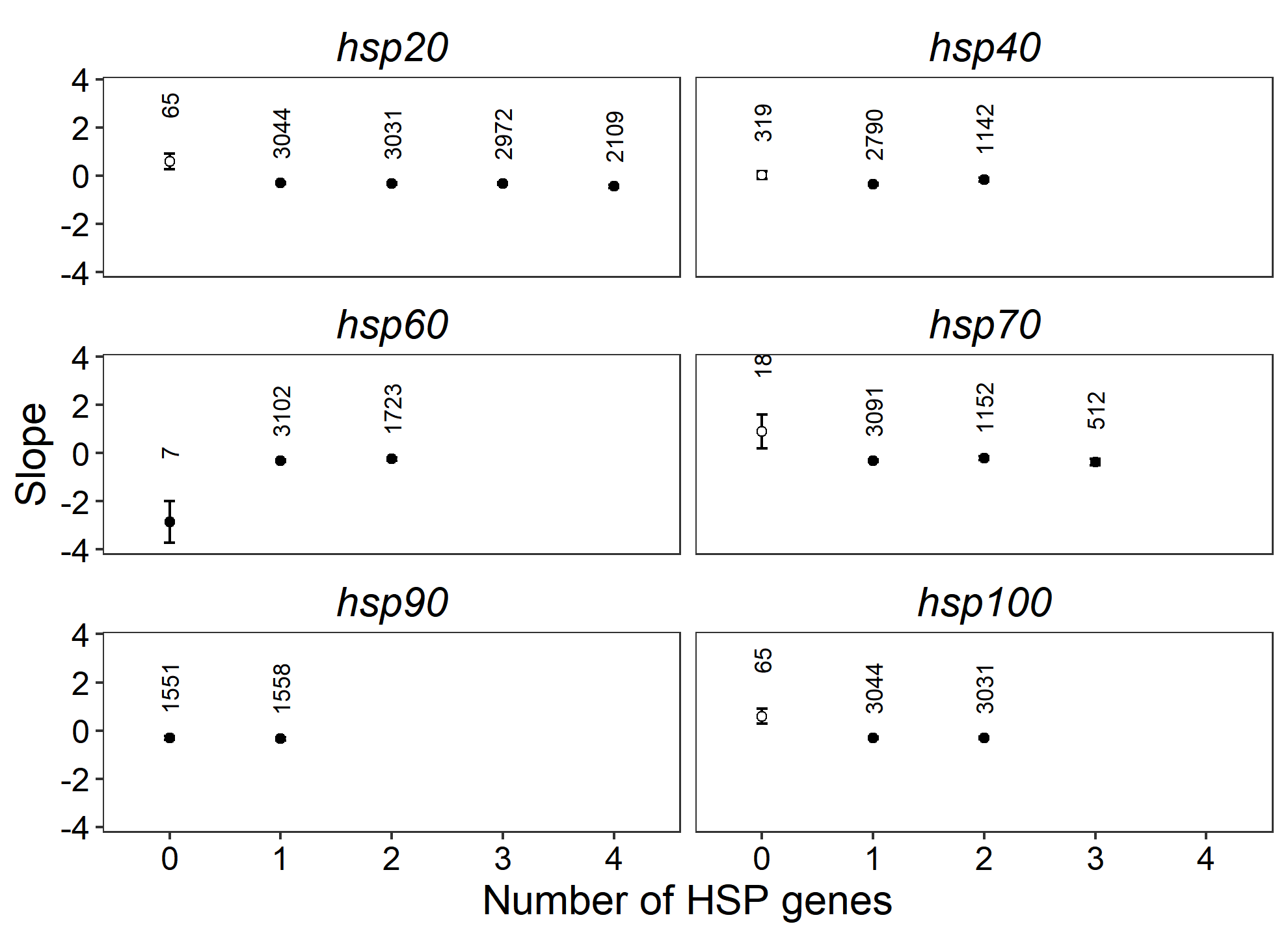


**Figure S9.** **The GrSR slopes across the number of genes encoding heat shock proteins (HSP)**. Numerical labels represent the number of genomes for analyses. Black points denote significant linear relationships (*P*_adj_ < 0.05), while grey points are nonsignificant. Error lines denote standard error.


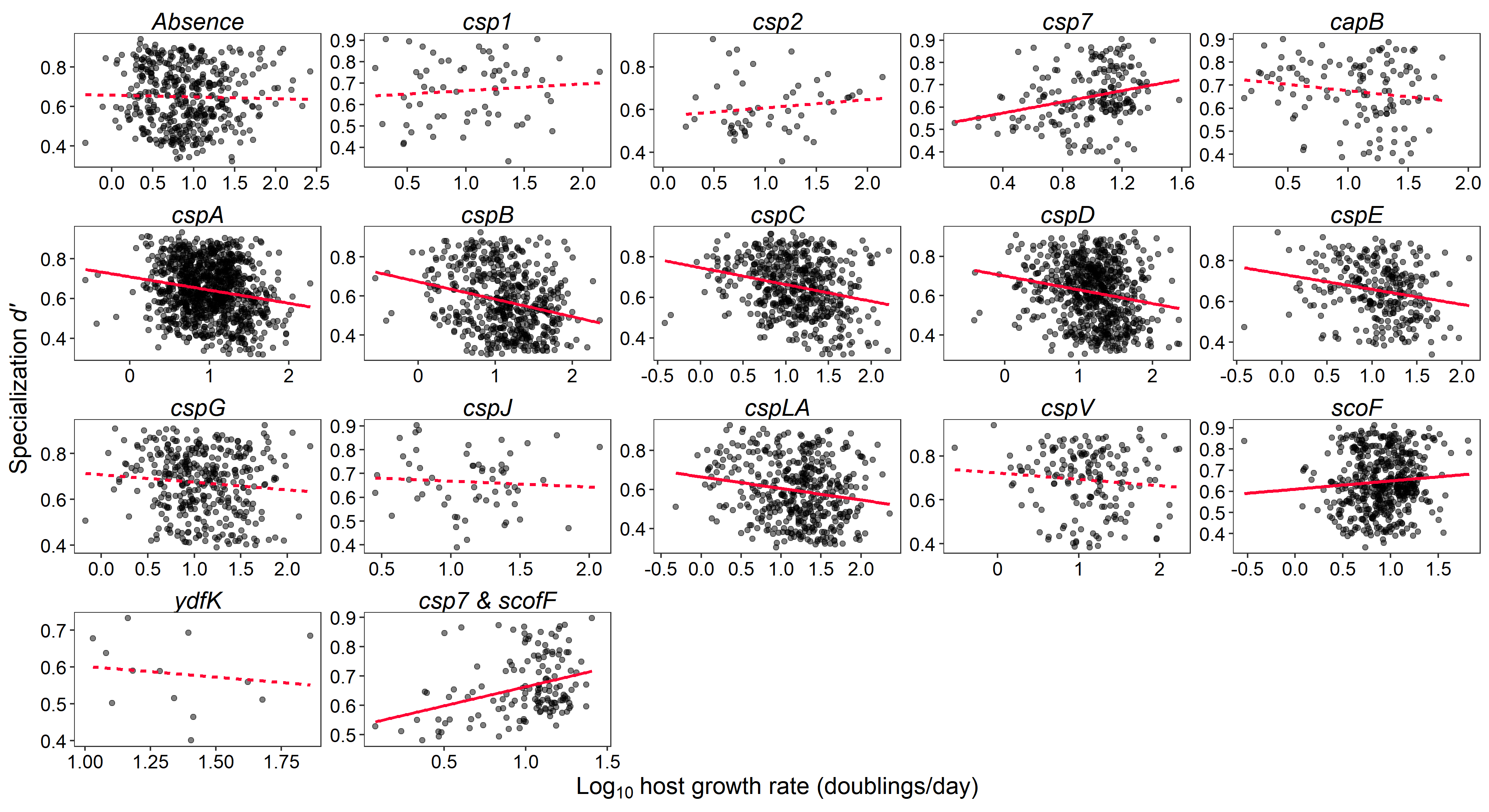


**Figure S10.** Effects of the genes encoding cold shock proteins (CSP) on GrSRs. Solid lines denote significant linear relationships (*P*_adj_ < 0.05), while dashed lines are nonsignificant.


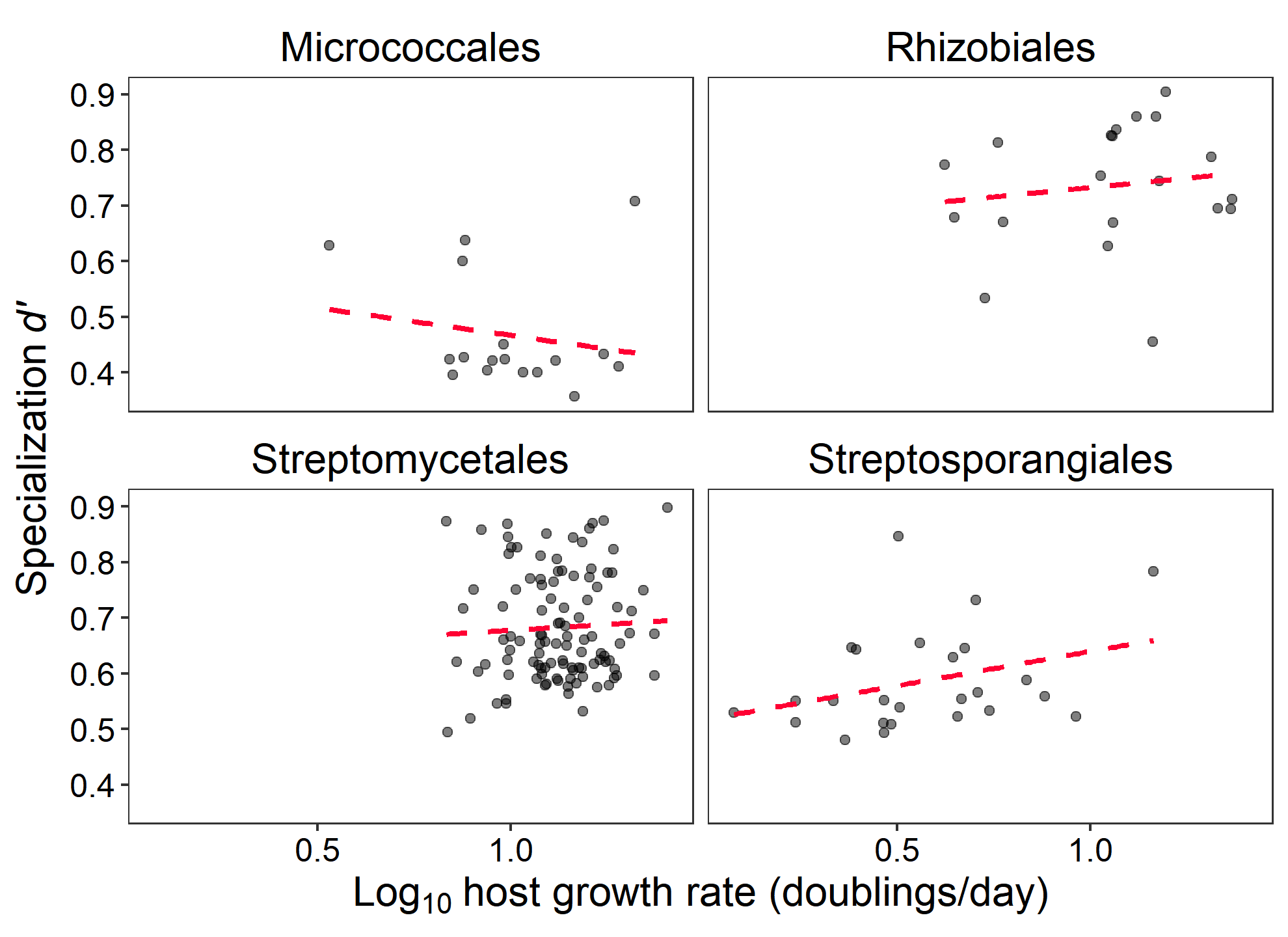


**Figure S11.** Effects of the *csp7* genes on GrSRs across main orders. Dashed lines denote nonsignificant linear relationships (all *P* > 0.1).


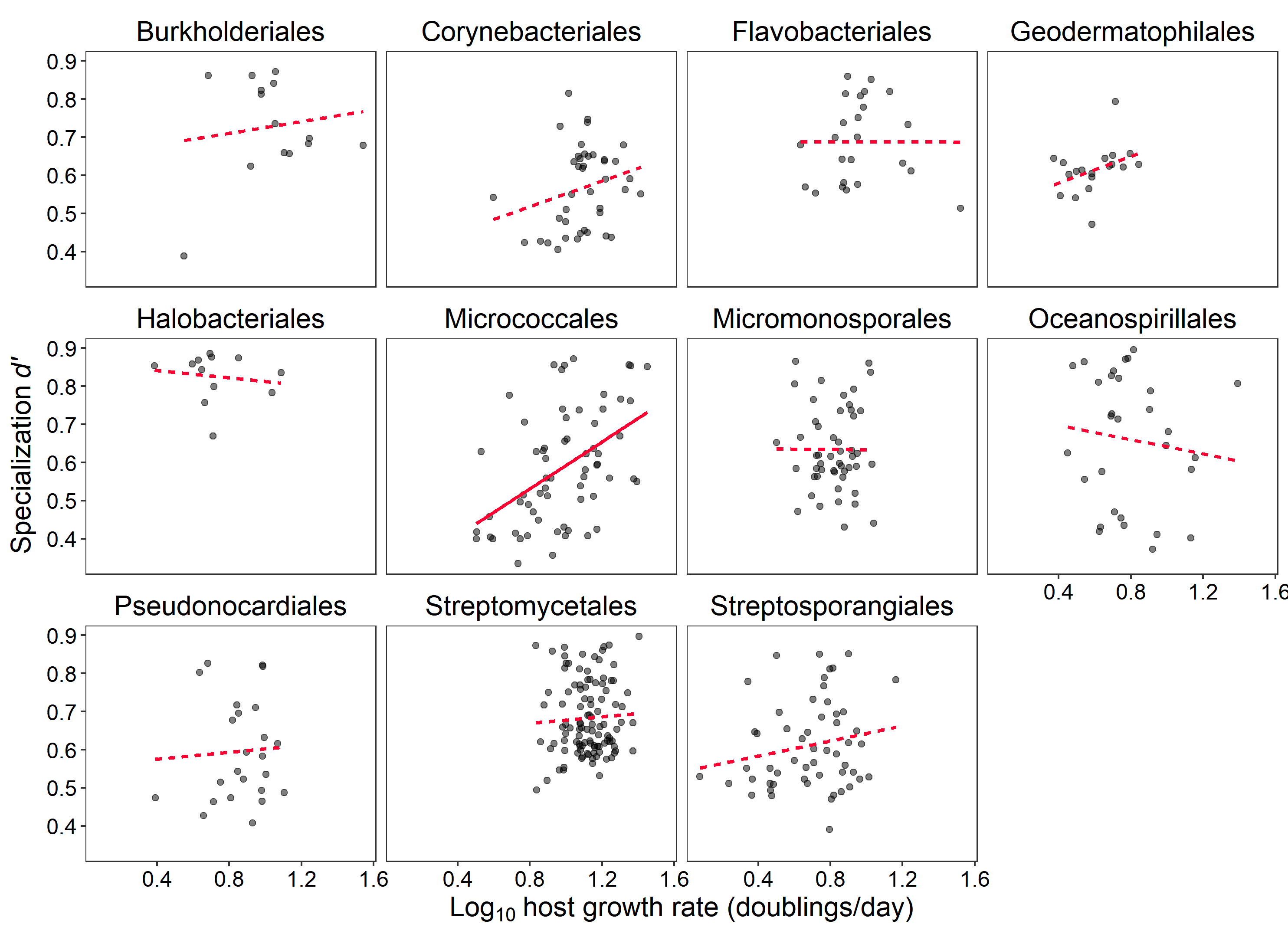


**Figure S12.** Effects of the *scoF* genes on GrSRs across various orders. Solid lines denote significant linear relationships (*P*_adj_ < 0.05), while dashed lines are nonsignificant.


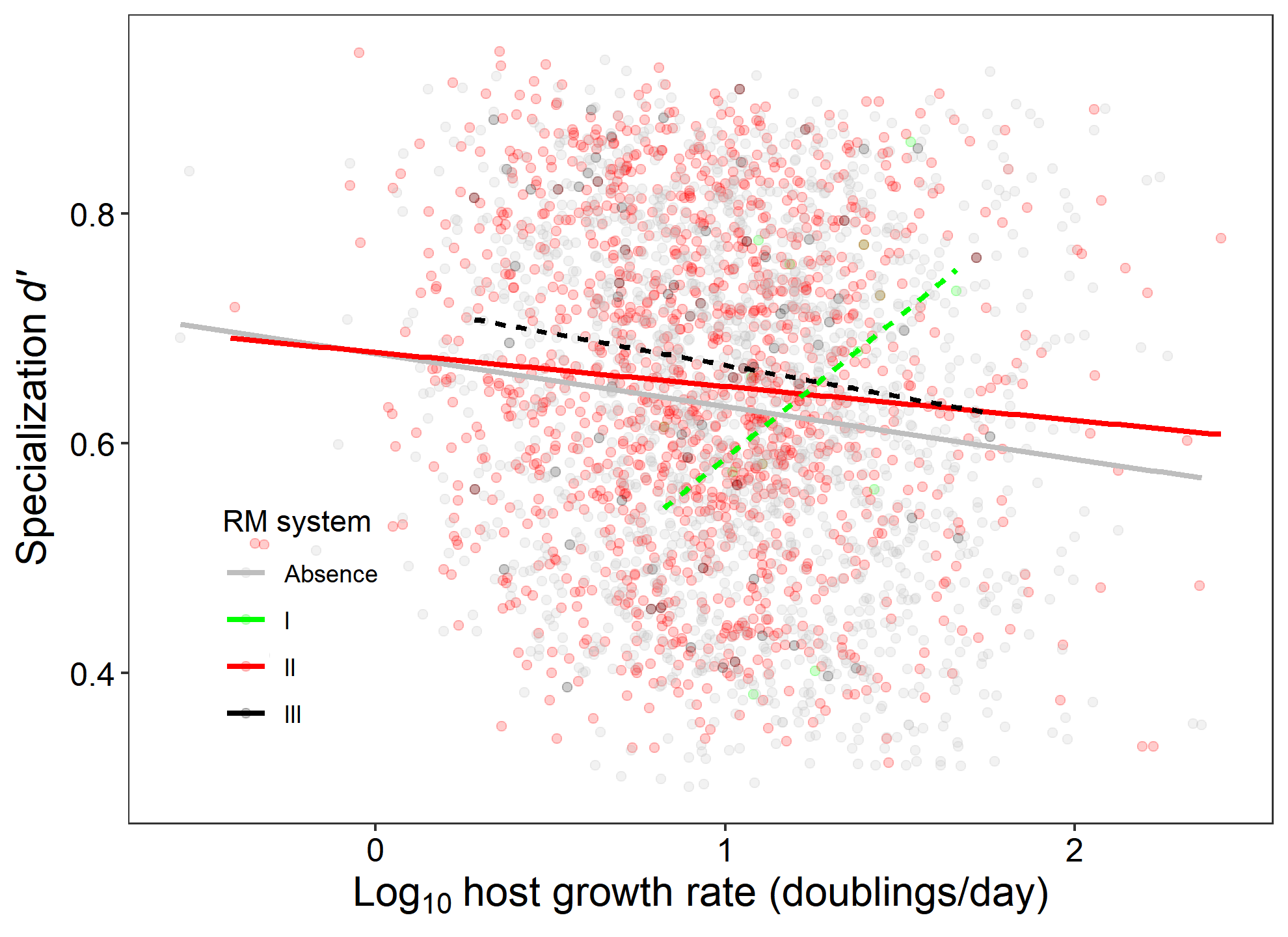


**Figure S13.** Effects of the RM systems on GrSRs. Solid lines denote significant linear relationships (*P*_adj_ < 0.05), while dashed lines are nonsignificant.


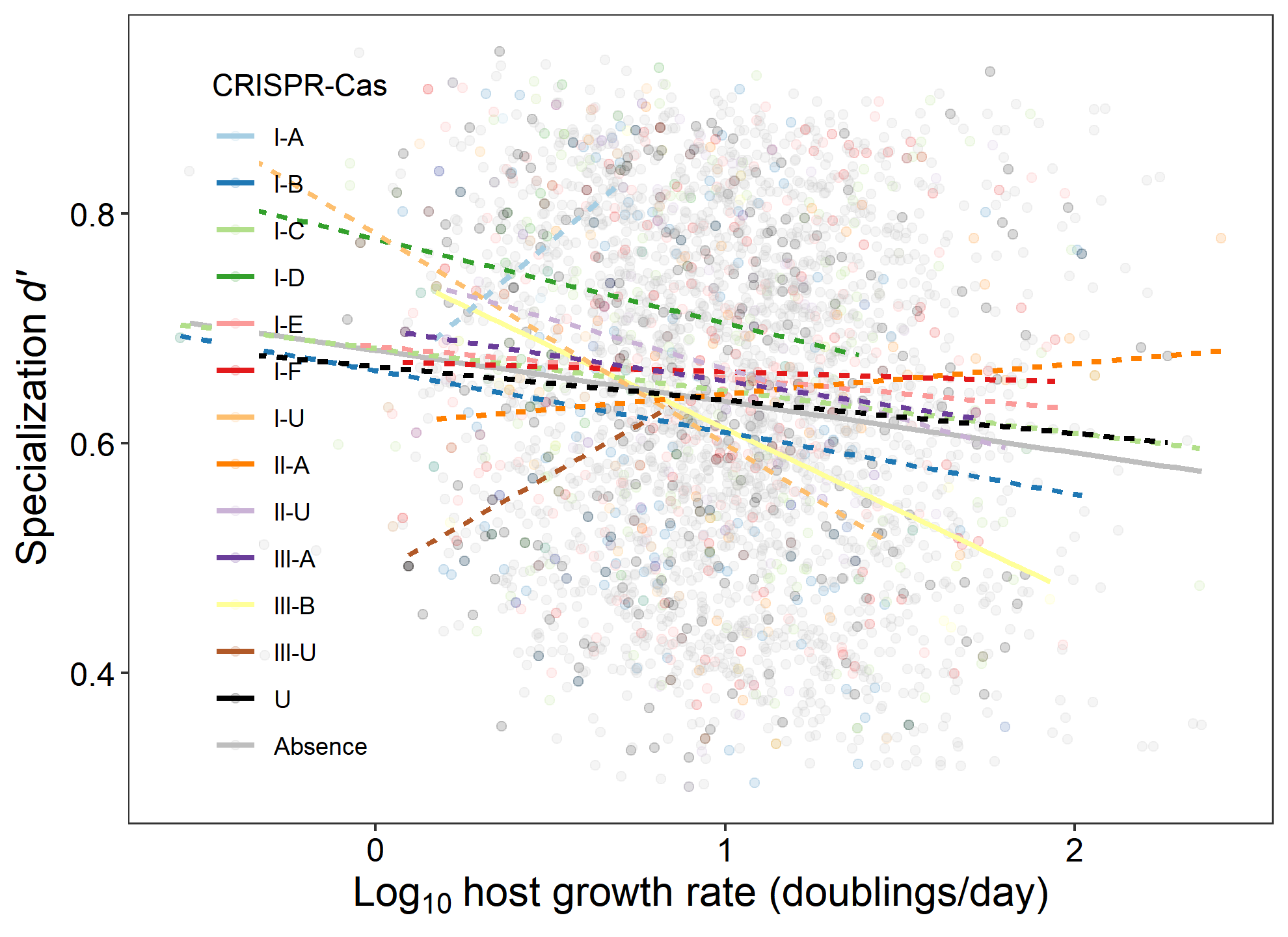


**Figure S14.** Effects of the CRISPR-Cas systems on GrSRs. Solid lines denote significant linear relationships (*P*_adj_ < 0.05), while dashed lines are nonsignificant.


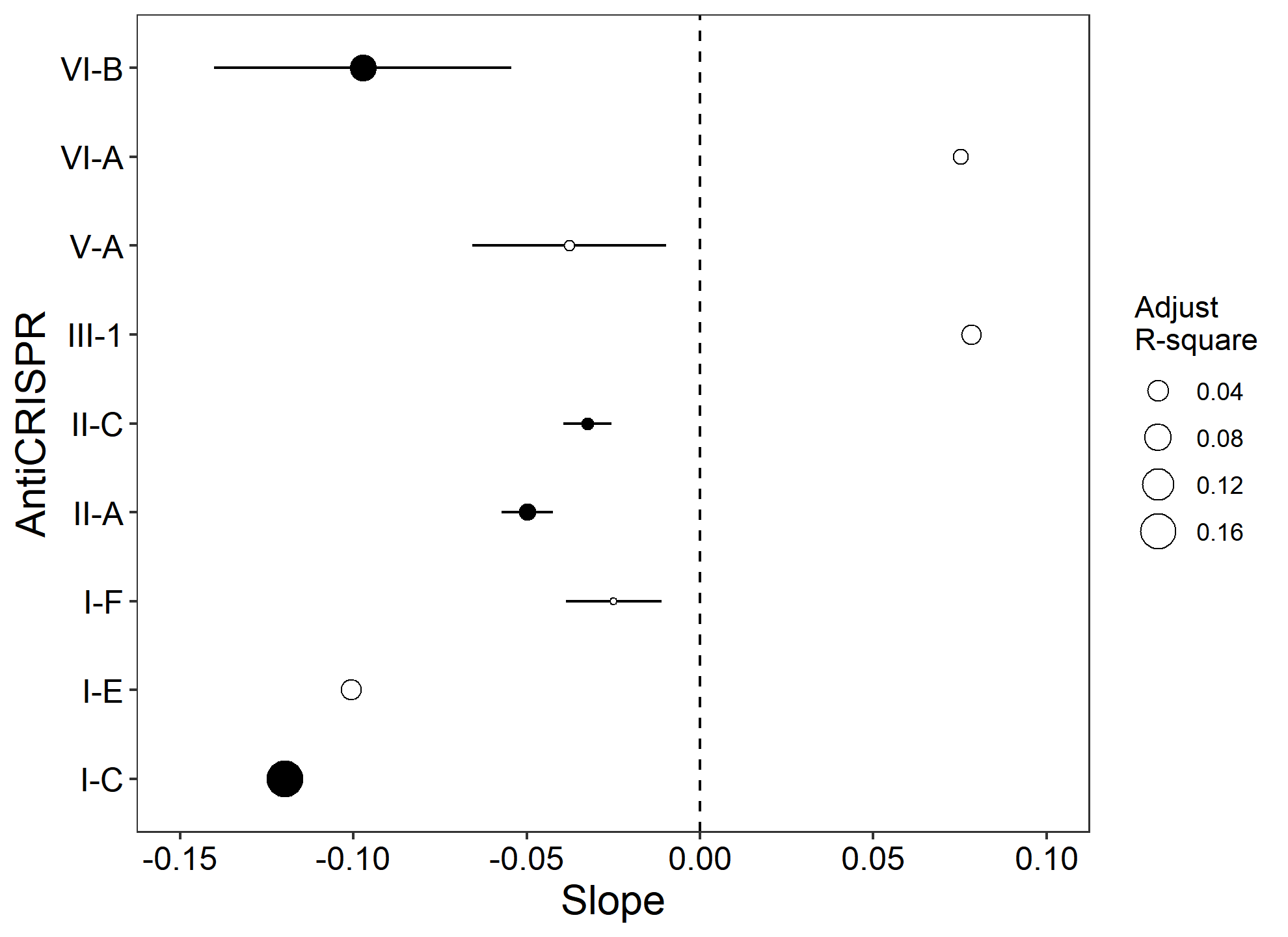


**Figure S15.** Effects of the anti-CRISPR systems on GrSRs. Solid lines denote significant linear relationships (*P*_adj_ < 0.05), while dashed lines are nonsignificant.


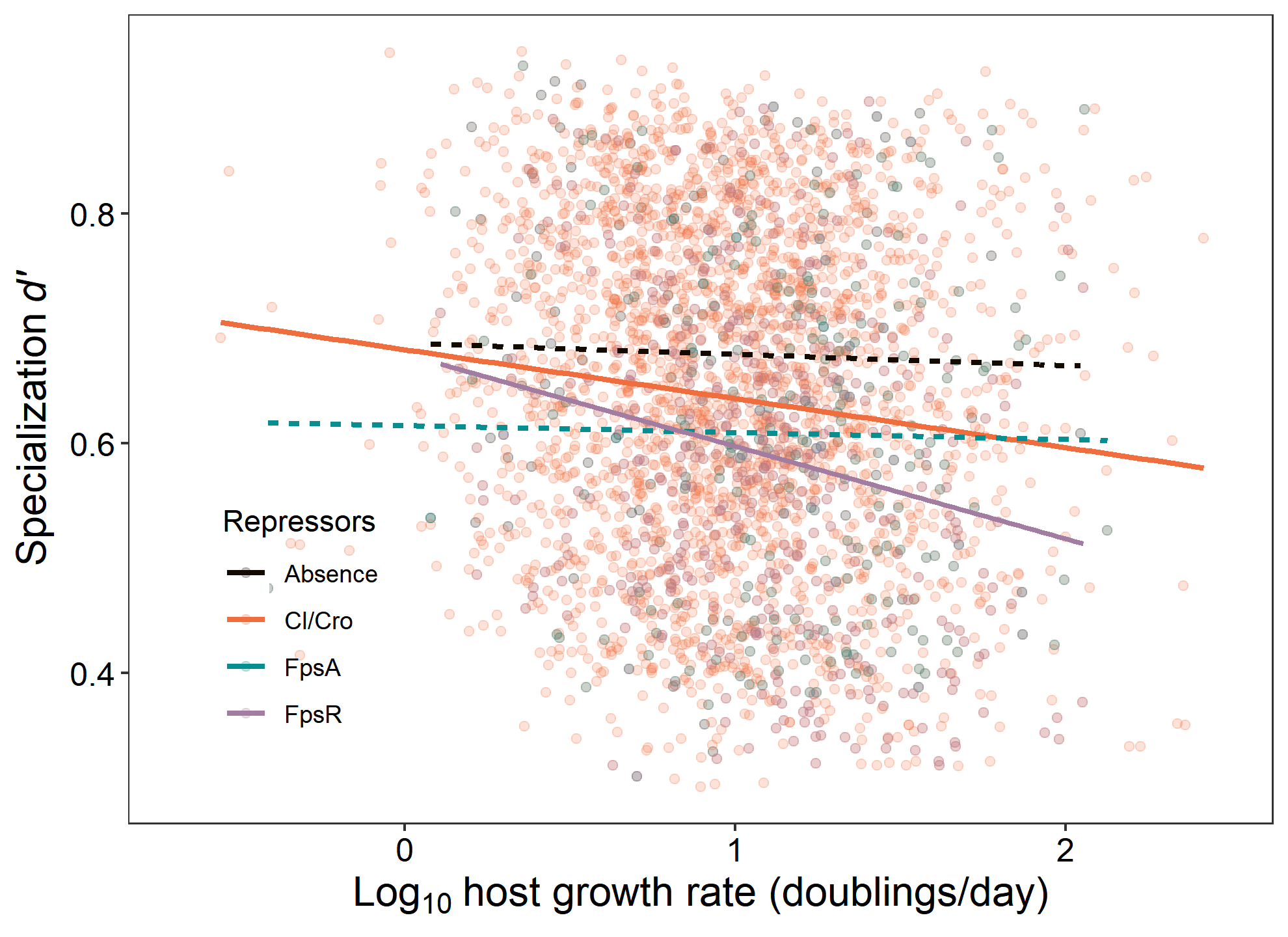


**Figure S16.** Effects of the temperature-dependent lytic switches on GrSRs. Solid lines denote significant linear relationships (*P*_adj_ < 0.05), while dashed lines are nonsignificant.


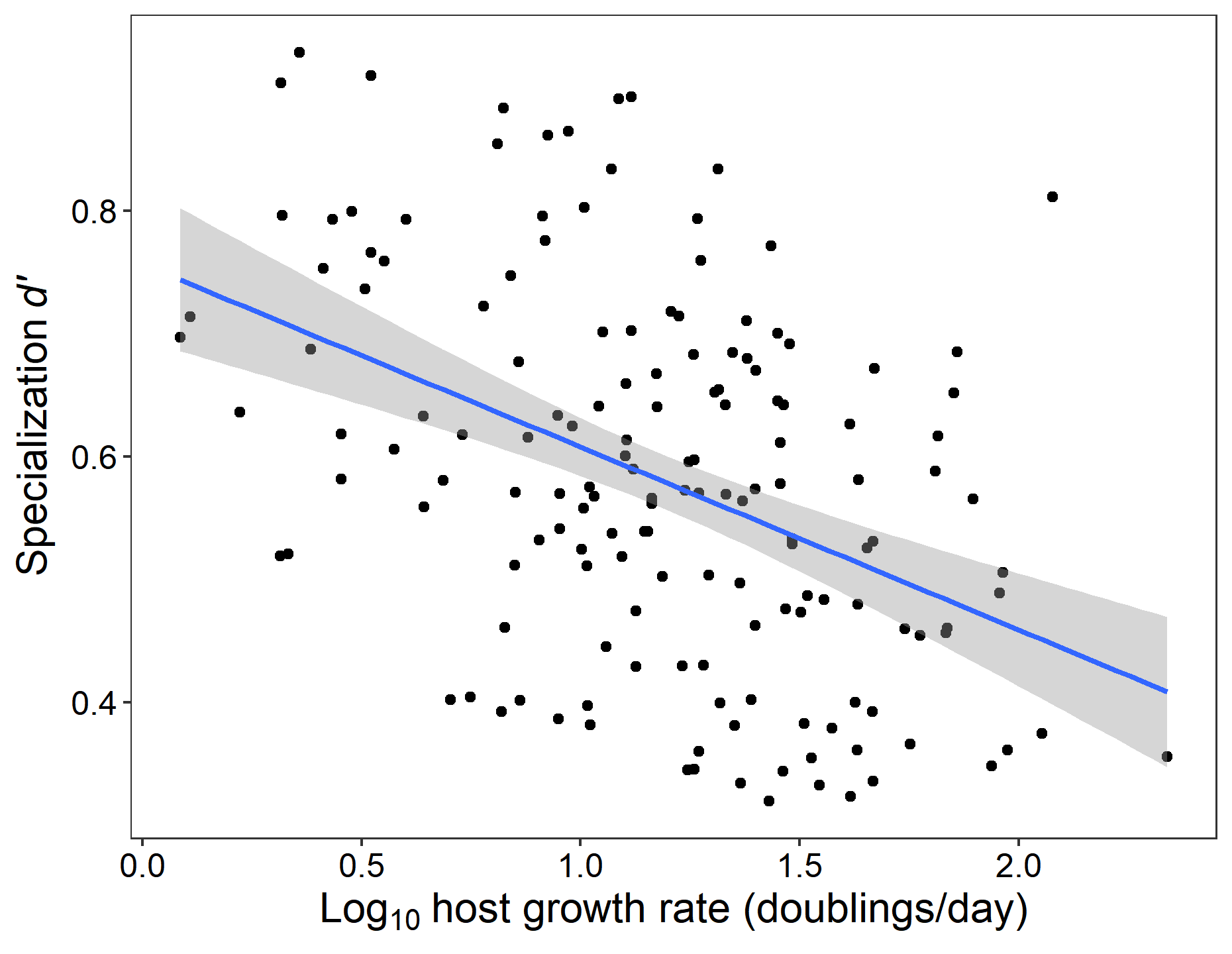


**Figure S17.** GrSRs for those species harboring the genes involving in the molecular processes in promoting the infection cycle at the stages of adsorption, establishment and viral release (R^2^_adj_ = 0.182, *P* < 0.001, df = 154). Those species harbor at least one gene encoding heat shock protein (*hsp20*, *hsp40*, *hsp70* or *hsp100*), at least two genes encoding cold shock protein (*cspC*, *cspD*, or *cspLA*), at least one gene encoding receptor protein (*fliC*, *fljB* or *mshA*), at least one pair of genes encoding heat lytic switch (*cI* and *cro*) and at least one gene encoding cold lytic switch (*fpsR*).


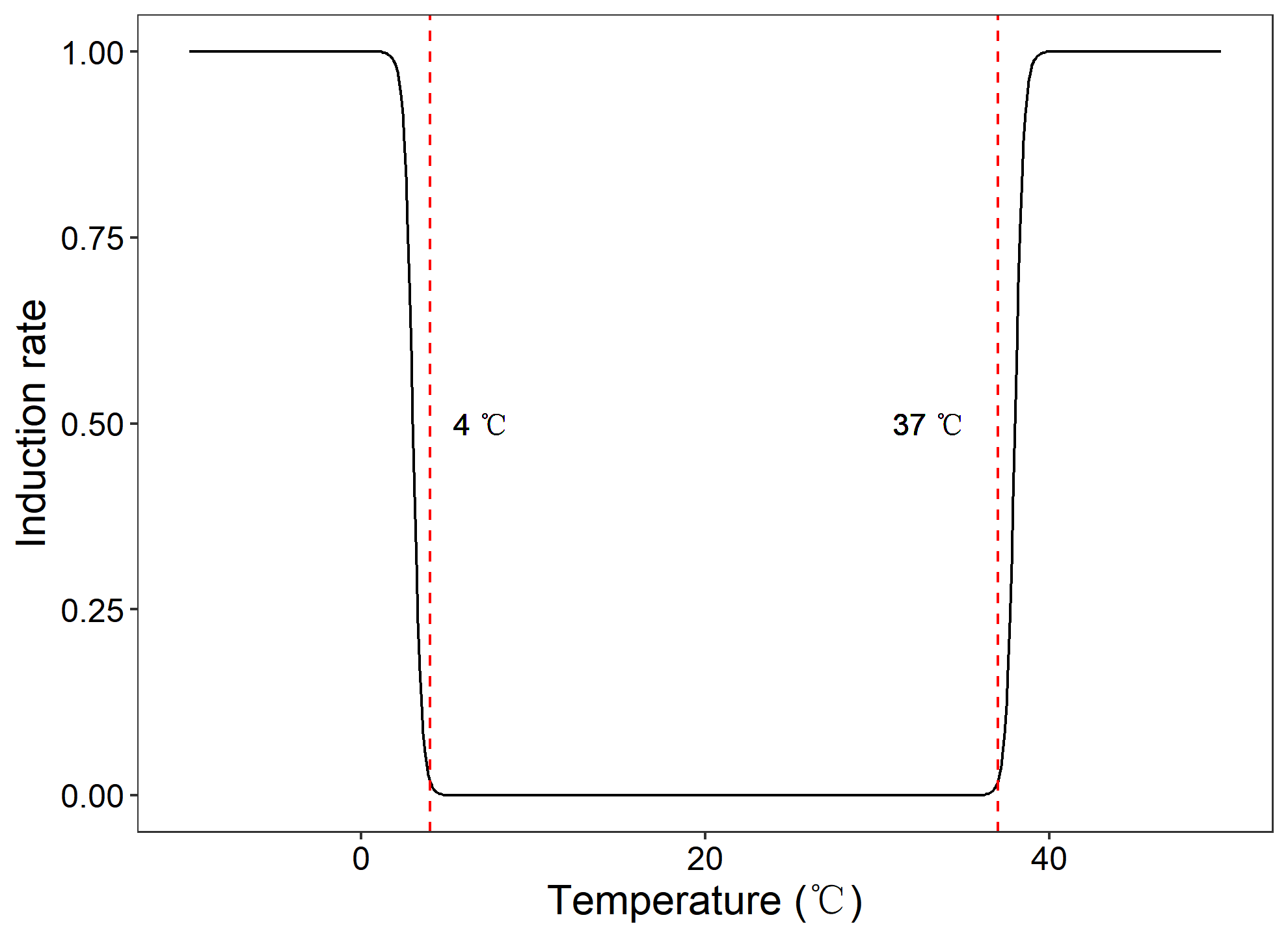


**Figure S18.** Temperature-dependent induction. It is controlled by two lytic switches including the repressors of CI/Cro (for high temperature at 37℃) and FpsR (for low temperature at 4 ℃), which is simulated by a Sigmond function.


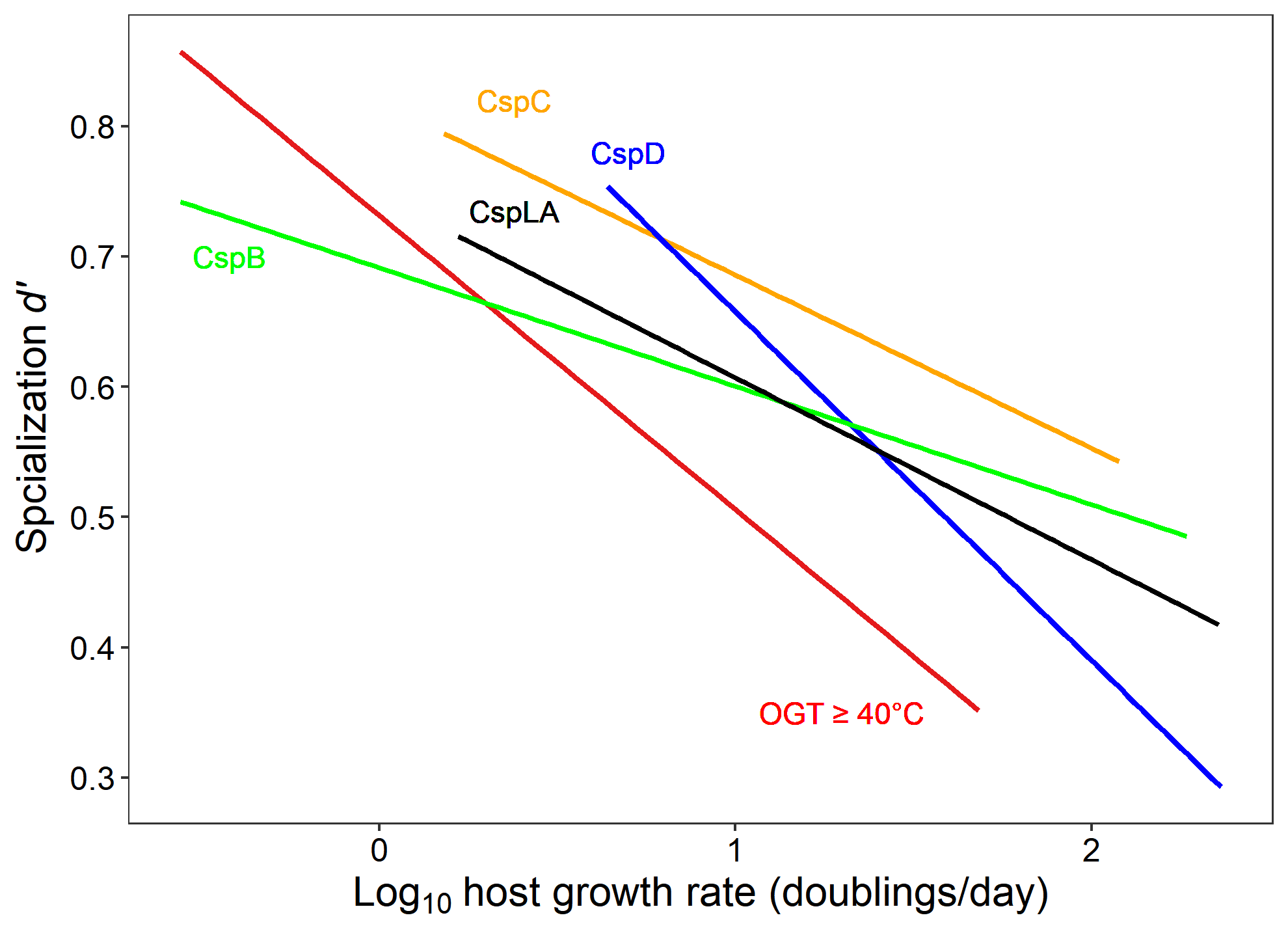


**Figure S19.** Significant linear GrSRs of the potential psychrophiles harboring at least three CSP genes and the thermophiles with OGT larger than 40℃. All *P*_adj_ < 0.05.

**Reference**

Bednarz, M., J. A. Halliday, C. Herman, and I. Golding. 2014. Revisiting bistability in the lysis/lysogeny circuit of bacteriophage lambda. PLoS One 9:e100876.

Egilmez, H. I., A. Y. Morozov, M. R. J. Clokie, J. Shan, A. Letarov, and E. E. Galyov. 2018. Temperature-dependent virus lifecycle choices may reveal and predict facets of the biology of opportunistic pathogenic bacteria. Sci Rep 8:9642.

Meng, C., S. Li, Q. Fan, R. Chen, Y. Hu, X. Xiao, and H. Jian. 2020. The thermo-regulated genetic switch of deep-sea filamentous phage SW1 and its distribution in the Pacific Ocean. FEMS Microbiol Lett 367.

Moyer, C. L., and R. Y. Morita. 2007. Psychrophiles and Psychrotrophs.

Pleska, M., M. Lang, D. Refardt, B. R. Levin, and C. C. Guet. 2018. Phage-host population dynamics promotes prophage acquisition in bacteria with innate immunity. Nat Ecol Evol 2:359-366.

Revell, L. J. 2010. Phylogenetic signal and linear regression on species data. Methods in Ecology and Evolution 1:319-329.

Revell, L. J. 2012. phytools: an R package for phylogenetic comparative biology (and other things). Methods in Ecology and Evolution:217-223.
